# Homoeolog expression bias in allopolyploid oleaginous marine diatom *Fistulifera solaris*

**DOI:** 10.1186/s12864-018-4691-0

**Published:** 2018-05-04

**Authors:** Tatsuhiro Nomaguchi, Yoshiaki Maeda, Tomoko Yoshino, Toru Asahi, Leila Tirichine, Chris Bowler, Tsuyoshi Tanaka

**Affiliations:** 1 Department of Advanced Science and Engineering, Graduate School of Advanced Science and Engineering, Waseda University, 3-4-1 Okubo, Shinjuku-ku, Tokyo, 169-8555 Japan; 2 Division of Biotechnology and Life Science, Institute of Engineering, Tokyo University of Agriculture and Technology, 2-24-16, Naka-cho, Koganei, Tokyo, 184-8588 Japan; 3 Ecole Normale Supérieure, PSL Research University, Institut de Biologie de l’Ecole Normale Supérieure (IBENS), CNRS UMR 8197, INSERM U1024, 46 rue d’Ulm, F-75005 Paris, France

**Keywords:** Allopolyploidy, *Fistulifera solaris* JPCC DA0580, Diatom, Pseudo-parental subgenome, Homoeolog expression bias

## Abstract

**Background:**

Allopolyploidy is a genomic structure wherein two or more sets of chromosomes derived from divergent parental species coexist within an organism. It is a prevalent genomic configuration in plants, as an important source of genetic variation, and also frequently confers environmental adaptability and increased crop productivity. We previously reported the oleaginous marine diatom *Fistulifera solaris* JPCC DA0580 to be a promising host for biofuel production and that its genome is allopolyploid, which had never previously been reported in eukaryotic microalgae. However, the study of allopolyploidy in *F. solaris* was hindered by the difficulty in classifying the homoeologous genes based on their progenitor origins, owing to the shortage of diatom genomic references.

**Results:**

In this study, the allopolyploid genome of *F. solaris* was tentatively classified into two pseudo-parental subgenomes using sequence analysis based on GC content and codon frequency in each homoeologous gene pair. This approach clearly separated the genome into two distinct fractions, subgenome Fso_h and Fso_l, which also showed the potency of codon usage analysis to differentiate the allopolyploid subgenome. Subsequent homoeolog expression bias analysis revealed that, although both subgenomes appear to contribute to global transcription, there were subgenomic preferences in approximately 61% of homoeologous gene pairs, and the majority of these genes showed continuous bias towards a specific subgenome during lipid accumulation. Additional promoter analysis indicated the possibility of promoter motifs involved in biased transcription of homoeologous genes. Among these subgenomic preferences, genes involved in lipid metabolic pathways showed interesting patterns in that biosynthetic and degradative pathways showed opposite subgenomic preferences, suggesting the possibility that the oleaginous characteristics of *F. solaris* derived from one of its progenitors.

**Conclusions:**

We report the detailed genomic structure and expression patterns in the allopolyploid eukaryotic microalga *F. solaris*. The allele-specific patterns reported may contribute to the oleaginous characteristics of *F. solaris* and also suggest the robust oleaginous characteristics of one of its progenitors. Our data reveal novel aspects of allopolyploidy in a diatom that is not only important for evolutionary studies but may also be advantageous for biofuel production in microalgae.

**Electronic supplementary material:**

The online version of this article (10.1186/s12864-018-4691-0) contains supplementary material, which is available to authorized users.

## Background

Allopolyploidy involves the coexistence of two or more sets of chromosomes within an organism that are derived from the hybridization of divergent species. This merger of distinct nuclear genomes often induces differentiated phenotypes in the offspring with respect to the parents, which may be intermediate between the two [1] or may induce novel phenotypic characteristics [2]. A rich history of botanical studies has shown the phenomenon to be particularly prevalent in plants, where it is considered as an important source of genetic variation driving plant speciation and that can also be exploited for crop improvement in agriculture. In particular, allopolyploid organisms frequently possess traits that render the organism advantageous for agricultural use, such as broad environmental adaptability [3–7] and high yields [6, 8]. Therefore, understanding the molecular mechanisms of genome regulation within allopolyploids is not only important in evolutionary and genetic contexts but also in agricultural and industrial settings. Many allopolyploid lineages of higher plants, including *Arabidopsis* [9–12], *Gossypium* (cotton) [13–15], *Triticum* (wheat) [16, 17], *Brassica* (oil seed) [18–20] and *Oryza* (rice) [21], have been studied since the concept of allopolyploidy was introduced by Kihara and Ono in 1926 [22].

Although allopolyploidy is prevalent in terrestrial plant lineages, allopolyploid eukaryotic microalgae have never been studied. Like plants, microalgae are a major group of eukaryotic photosynthetic organisms displaying enormous diversity, with estimates of up to several million species worldwide [23]. But besides their capacity for photosynthesis and being unicellular, there is little evolutionary commonality between microalgae because they have at least three distinct origins. Red and green algae both lie within the Archaeplastida lineage (like higher plants) yet diverged very early during the evolution of eukaryotes. Outside the archaeplastids, the major groups of microalgae are found in the “CASH lineages”, consisting of photosynthetic members of cryptomonads, alveolates (such as dinoflagellates), stramenopiles (including diatoms) and haptophytes [24–26]. Study of diversification and speciation is essential to inform us about the evolutionary processes that have led to such dramatically diversified groups of ecologically successful organisms. Furthermore, microalgae are also being studied in the context of biotechnology because they are recognized as promising hosts for the production of functional biomacromolecules, such as fatty acids, carotenoids, and phycocolloids, which are useful for various applications, including in healthcare, food and feed, pharmaceuticals, and biofuels [27]. By analogy with modern agriculture, it can also be expected that the large-scale cultivation processes used in industrial applications with microalgae could benefit from the availability of allopolyploid strains.

In the approximately century-long history of allopolyploid research, our group discovered the first allopolyploid microalga, the diatom *Fistulifera solaris* JPCC DA0580 [28, 29]. *F. solaris* is a pennate diatom isolated from river mouths in Kagoshima, Japan. It has a high neutral lipid content [30], sufficient growth characteristics in large-scale outdoor cultivation systems [31, 32], and the ability to accumulate neutral lipids while growing in logarithmic phase [29], which are all beneficial features for its use in biofuel production. We also recently determined its whole genome sequence using next-generation sequencing technology [29], and noted that the *F. solaris* genome was almost double the size of the other two sequenced model diatoms, *Phaeodactylum tricornutum* [33] and *Thalassiosira pseudonana* [34], and contains approximately twice the number of protein coding genes. Subsequent sequence analysis suggested that its genome is allopolyploid, consisting of 42 homoeologous chromosome pairs with 75% overall sequence homology, and containing two distinct types of 18S rRNA and 9007 pairs of duplicated genes, or more commonly stated, homoeologous genes or homoeologues [29]. A similar genomic structure was also reported for the genome of the cold-adapted diatom *Fragilariopsis cylindrus* [35]; however, this divergence was only observed within 24.5% of its total genome, and so it is unlikely to be an allopolyploid. To our knowledge *F. solaris* is the only microalga that displays evidence for allopolyploidy. Conversely, natural intraspecies hybrids of diatoms have only been reported between *Pseudo-nitzschia pungens* var. *pungens* and var. *cingulata* [36], and while artificial interspecies hybridization leading to allopolyploidy may be feasible under laboratory conditions, its low success rates and limited species distribution obstruct further study [37]. We therefore consider that *F. solaris* could be used as a model organism to study the molecular mechanisms underlying allopolyploidy in unicellular microalgae.

Study of allopolyploidy in *F. solaris* can contribute insights into the evolutionary history of genome structure in diatoms, as well as the genetic underpinnings of the oleaginous phenotype of this diatom, which is beneficial for biofuel production. However, a challenge to be overcome to proceed with this study is the difficulty in obtaining the reference genomes of progenitor species. Most studies on allopolyploidy [7, 9, 10, 13, 14, 17] have used model allopolyploid plants for which high quality reference genomes are available from the progenitor species. This enables a comparison of the gene expression profiles of the target allopolyploid species and that of its progenitors, which provides information as to whether the homoeologous genes are expressed additively or non-additively compared with the parent plants [14, 17, 38], thus allowing evaluation of the relationships between expression patterns and phenotypes within the allopolyploids. However, since genome data for microalgae is not widely available in global databases, it is difficult to obtain and validate parental genome data from such organisms.

In the present study, we first attempted to constrain the genome of *F. solaris* into two pseudo-parental subgenomes, which we considered to be derived from each progenitor based on GC content ratio (dGC) and codon usage bias. Interestingly, the higher-dGC member of each pair of homoeologous genes was localized to one of the homoeologous chromosomes, and the lower-dGC member of the pair was localized on the other. In addition, the correlation of codon usage bias between chromosomes with high-dGC homoeologous genes and low-dGC homoeologous genes was well conserved in the majority of chromosome pairs, suggesting that the comparison of codon usage bias may have potency to differentiate the allopolyploid subgenome. Classification of differential expression in homoeologous gene pairs was analysed genome-wide and for some specific metabolic pathways in order to obtain insights into the impacts of allopolyploidy on the metabolism of *F. solaris*. In particular, the relationship between allopolyploidy and lipid metabolism was examined. Although genome-wide mRNA abundance analysis failed to reveal significant biases towards specific subgenomes, homoeologous genes with high expression levels showed preferential expression towards the high-dGC subgenome. In contrast to this global preference, we observed that the homoeologous genes involved in fatty acid and lipid biosynthesis showed preferential expression towards the low-dGC subgenome. These outcomes suggested that while both subgenomes of *F. solaris* retain some expression, their expression ratio is not equal, suggesting that the combination of unequally expressing subgenomes may contribute to its characteristics, and that the differential expression of a specific subgenome encoding lipid metabolism might contribute to the high oil accumulation characteristics of this diatom.

## Results

### Classifying homoeologous gene pairs into two subgenomes

In the previous study, 9007 pairs of homoeologous genes and 2441 of non-homoeologous gene, which only exist in one of the homoeologous chromosome pair, were annotated [29]. However, the origin of each pair remained unclear. Classifying these genes without reference genome, we adopted two strategies based on species-specific sequence analysis that do not require additional sequence information. In the first strategy, we compared the dGC of each homoeologous gene of a homoeologous chromosome pair, based on which Louis et al. have demonstrated the classification of the genome of an allopolyploid yeast into two parental subgenomes [39]. We then analysed the codon usage bias of homoeologous genes in each homoeologous chromosome pair by principal component analysis (PCA) as a second strategy.

**Fig. 1:**
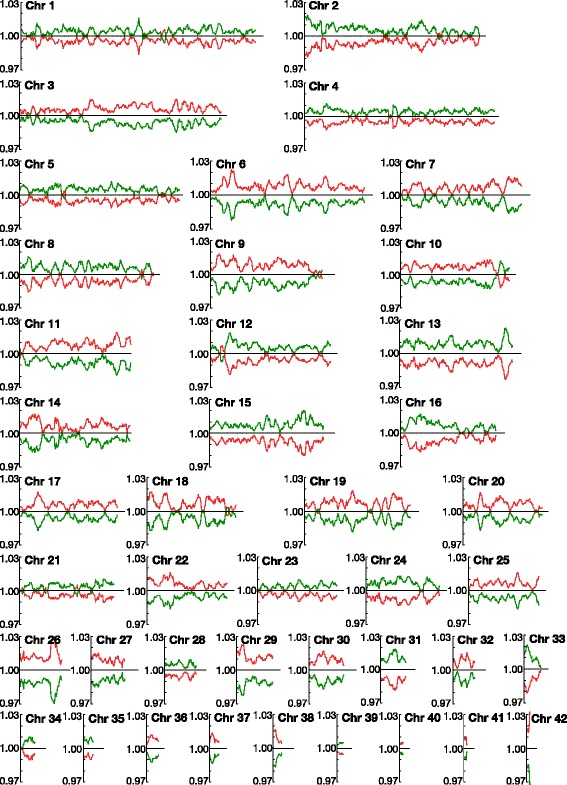
Comparison of GC content between pairs of homoeologous genes in each homoeologous chromosome. Vertical axis represents GC content ratio (dGC) value of each homoeologous gene, and horizontal axis indicates the relative number of homoeologous genes on each chromosome. The dGC value indicates the ratio of GC content in one homoeologous gene with respect to its corresponding pair. As such, if the dGC value is over 1 (above the horizontal axis), its GC content is higher than the other pair. The dGC value of homoeologous genes along the homoeologous chromosome with higher global GC content (Chromosome X) and lower global GC content (Chromosome X’) were indicated as red and green line, respectively

We calculated and compared dGC of every homoeologous gene and its syntenic copy coded along paired chromosomes for all 42 chromosome pairs (Fig. 1). As shown in Fig. 1, homoeologous genes with relatively high dGC and low dGC were clearly separated and localised in each paired chromosome. Approximately half of homoeologous chromosome pairs showed that homoeologous chromosome with lower GC content contain high-dGC homoeologous genes, represented by swapped colour in Fig. 1, suggesting the low correlation between chromosome global GC content and dGC. We hypothesised that this separation was caused by differences in nucleotide composition that originated from each progenitor. The only exception was chromosome pair 10, where the reverse dGC relationship between the pairs was observed in the 3′ terminal segment. Unlike the other brief reciprocal reversal of the dGC trends observed at several points in other chromosomes, this exchange continued over a broad region (ca. 220 kilobases), and the difference in dGC ratio was also distinctive. These data suggest that, unlike the other small exchanges which we believe to correspond to noise, the reversal of the dGC trend observed on chromosome pair 10 could be caused by intergenomic rearrangements such as crossing over following interspecies hybridization, as has been reported for allodiploid yeast [39]. To examine the possibility of such rearrangements in more detail, we confirmed the scaffold assembly of the corresponding region in chromosome 10′, which consisted of a single long scaffold, by polymerase chain reaction and electrophoresis (Additional file 1: Figure S1B, C). We also confirmed the local dGC distribution around the 3′-terminal segment, and confirmed that the dGC trend was reversed at the fso:g7228/fso:g15955 pair and that this reversed trend continued through the downstream region (Additional file 1: Figure S1A). Based on these results, we tentatively classified the chromosomes with high dGC as belonging to pseudo-parental subgenome Fso_h and the other chromosome counterpart as belonging to pseudo-parental subgenome Fso_l, except for chromosome 10. For the homoeologous pair of chromosome 10, the regions with relatively higher dGC were designated as belonging to Fso_h and their counterparts to Fso_l. Such exchange of dGC among the chromosome pair was not evident from global GC content analysis of each chromosome that was reported in our previous study [29], and thus we emphasize the dGC analysis of *F. solaris* genome as one of the novelties of the present study.

To support the classification based on dGC comparison, we also compared the codon usage frequencies in the tentatively classified subgenomes (Additional file 2: Table S1 and Additional file 3: Figure S2). In general, the global frequencies of Fso_h and Fso_l were more similar to each other than to other sequenced diatoms (Additional file 3: Figure S2A). However, some biases were observed; for instance, among the synonymous codons encoding leucine, Fso_h used UUG more frequently than Fso_l, whereas Fso_l used CUU more frequently than Fso_h. When the ratios of codon usage frequency of the Fso_h and Fso_l subgenomes were compared, A or U was used more frequently at the third positions of the codons in Fso_l than in Fso_h; In contrast, G or C was used more frequently by Fso_h than by Fso_l (Additional file 3: Figure S2B, C). Moreover, dGC differed more between the two subgenomes at the third codon position (49.2% for Fso_h and 47.9% for Fso_l) than at the first and second codon positions (Additional file 2: Table S1B). To examine whether this tendency was maintained throughout the chromosomes, the dGC specifically at the third codon position (dGC3) was plotted in a manner similar to the global dGC shown in Fig. 1. Overall the dGC3 prolife (Additional file 4: Figure S3) was perfectly consistent with the dGC profile, including the reversal of the dGC3 trend in the chromosome 10 pair. This result indicates that the observed trends in the third codon position support the classification of subgenomes based on dGC.

**Fig. 2:**
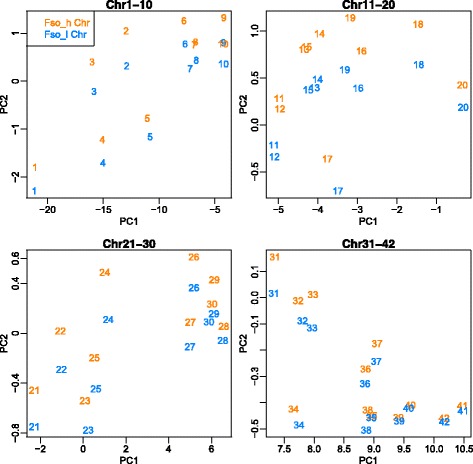
PCA based on codon usage frequency of homoeologous genes in each chromosome. The figure shows the two dimensional principal component analysis (PCA) results based on first principal component (PC1) and second principal component (PC2). Each plotted number represents a chromosome number and the colour corresponds to the subgenome to which they belong

As a further analysis of the codon usage, we incorporated the statistical analysis to examine the global bias in distribution pattern of codon usage between two subgenomes, expressed as codon usage bias. The frequency of the 64 variations of codons used in homoeologous genes was calculated for every chromosome, and PCA was computed. The result was plotted in a biplot with PC1 and PC2 (Fig. 2). Each chromosome number was plotted distantly in the biplot in PC1 direction, and each pair of homoeologous chromosomes was vertically positioned (PC2). Strikingly, all chromosome pairs separated into homoeologous chromosomes in the direction of PC2, with chromosome with higher dGC having more positive PC2 values. Notably, the codon frequency bias of the 3′ terminal region of chromosome 10, where the reversal in dGC trend was observed, also showed the same trend, which supports the possibility of an instance of crossing over having occurred (Additional file 5: Figure S4). Thus, PCA biplots showing codon usage patterns also support the classification of subgenomes based on dGC.

We also calculated the loadings during the PCA in order to estimate which codons most strongly contribute to each principal component (Additional file 2: Table S2). All the loadings in PC1 were negative, and were distributed in a small range between − 0.965 and − 0.999. Therefore, it is likely that there were no significant differences in the intensity of the contribution of each codon. The differences in PC1 score originate from the variation in the genes contained on each chromosome. In contrast, the PC2 loadings ranged between 0.161 and − 0.191. The relatively large differences in values compared to those of PC1 suggest that codon usage bias contributed more strongly to PC2. For instance, synonymous codon variation for leucine showed the large difference in PC2, which is consistent with the result presented in Additional file 3: Figure S2. We thus conclude that codon usage bias strongly contributed to PC2, which caused a large separation of homoeologous chromosomes in the vertical direction.

Another point worth noting is that in 38 out of 42 pairs of homoeologous chromosomes, a greater number of non-homoeologous genes was predicted on the chromosomes with high dGC homoeologous genes (Table 1). In the chromosome 10/10′ pair (the chromosomes with relatively high/low GC content), this relationship was also reversed at the 3′ terminal region where the reversal of the dGC trend was observed. The count of non-homoeologous genes revealed that the high-dGC region within chromosome 10′ contained twice as many non-homoeologous genes than those in the syntenic low-dGC region within chromosome 10. This confirms the general trend that the high-dGC subgenome contains more non-homoeologous genes.

**Table 1:** Chromosome characteristics of allopolyploid diatom *F. solaris*

Chromosome	Global GC content		Number of predicted genes		Number of homoeologous genes		Number of non-homoeologous genes	
	H^a^	L^a^	H	L	H	L	H	L
Chr 1	45.27	45.31	692	649	617 (89.16)^b^	617 (95.07)	75 (10.84)	32 (4.93)
Chr 2	45.34	45.58	585	519	463 (79.15)	463 (89.21)	122 (20.85)	56 (10.79)
Chr 3	45.93	45.77	595	562	531 (89.24)	531 (94.48)	64 (10.76)	31 (5.52)
Chr 4	45.24	45.30	561	547	499 (88.95)	499 (91.22)	62 (11.05)	48 (8.78)
Chr 5	44.85	45.04	497	454	421 (84.71)	421 (92.73)	76 (15.29)	33 (7.27)
Chr 6	45.82	45.81	452	422	408 (90.27)	408 (96.68)	44 (9.73)	14 (3.32)
Chr 7	45.82	45.68	384	345	322 (83.85)	322 (93.33)	62 (16.15)	23 (6.67)
Chr 8	45.59	45.65	387	382	354 (91.47)	354 (92.67)	33 (8.53)	28 (7.33)
Chr 9	45.75	45.36	364	340	299 (82.14)	299 (87.94)	65 (17.86)	41 (12.06)
Chr 10	45.21	44.83	358	364	291 (81.28)	291 (79.95)	67 (18.72)	73 (20.05)
Chr10_A^c^	/	/	308	285	256 (83.11)	256 (89.82)	52 (16.88)	29 (10.18)
Chr10_B^c^	/	/	79	50	35 (44.30)	35 (70.00)	44 (55.70)	15 (30.00)
Chr 11	45.89	45.83	343	314	297 (86.59)	297 (94.59)	46 (13.41)	17 (5.41)
Chr 12	45.75	46.12	381	343	320 (83.99)	320 (93.29)	61 (16.01)	23 (6.71)
Chr 13	45.17	45.39	356	332	301 (84.55)	301 (90.66)	55 (15.45)	31 (9.34)
Chr 14	45.91	45.64	360	332	293 (81.39)	293 (88.25)	67 (18.61)	39 (11.75)
Chr 15	45.50	45.51	338	326	303 (89.64)	303 (92.94)	35 (10.36)	23 (7.06)
Chr 16	45.81	46.14	307	271	255 (83.06)	255 (94.10)	52 (16.94)	16 (5.90)
Chr 17	46.41	45.81	294	288	260 (88.44)	260 (90.28)	34 (11.56)	28 (9.72)
Chr 18	45.73	45.48	291	263	242 (83.16)	242 (92.02)	49 (16.84)	21 (7.98)
Chr 19	46.22	45.78	310	297	282 (90.97)	282 (94.95)	28 (9.03)	15 (5.05)
Chr 20	45.95	45.73	263	234	214 (81.37)	214 (91.45)	49 (18.63)	20 (8.55)
Chr 21	45.17	45.40	273	265	252 (92.31)	252 (95.09)	21 (7.69)	13 (4.91)
Chr 22	46.02	45.94	247	238	218 (88.26)	218 (91.60)	29 (11.74)	20 (8.40)
Chr 23	45.07	45.42	250	231	215 (86.00)	215 (93.07)	35 (14.00)	16 (6.93)
Chr 24	45.81	45.82	250	212	199 (79.60)	199 (93.87)	51 (20.40)	13 (6.13)
Chr 25	45.38	45.21	216	203	191 (88.43)	191 (94.09)	25 (11.57)	12 (5.91)
Chr 26	45.65	44.56	165	130	118 (71.52)	118 (90.77)	47 (28.48)	12 (9.23)
Chr 27	46.11	45.32	107	118	96 (89.72)	96 (81.36)	11 (10.28)	22 (18.64)
Chr 28	44.58	45.34	119	101	92 (77.31)	92 (91.09)	27 (22.69)	9 (8.91)
Chr 29	46.38	45.38	129	114	103 (79.84)	103 (90.35)	26 (20.16)	11 (9.65)
Chr 30	45.60	45.45	121	117	102 (84.30)	102 (87.18)	19 (15.70)	15 (12.82)
Chr 31	45.36	45.63	89	78	74 (83.15)	74 (94.87)	15 (16.85)	4 (5.13)
Chr 32	45.88	45.71	79	73	67 (84.81)	67 (91.78)	12 (15.19)	6 (8.22)
Chr 33	44.92	46.05	63	57	54 (85.71)	54 (94.74)	9 (14.29)	3 (5.26)
Chr 34	46.24	46.36	54	50	47 (87.04)	47 (94.00)	7 (12.96)	3 (6.00)
Chr 35	44.42	44.99	43	40	35 (81.40)	35 (87.50)	8 (18.60)	5 (12.50)
Chr 36	46.20	46.16	50	47	42 (84.00)	42 (89.36)	8 (16.00)	5 (10.64)
Chr 37	46.48	46.42	45	34	34 (75.56)	34 (100.00)	11 (24.44)	0 (0.00)
Chr 38	46.93	45.68	34	37	33 (97.06)	33 (89.19)	1 (2.94)	4 (10.81)
Chr 39	45.55	45.80	33	27	27 (81.82)	27 (100.00)	6 (18.18)	0 (0.00)
Chr 40	45.58	45.42	25	21	19 (76.00)	19 (90.48)	6 (24.00)	2 (9.52)
Chr 41	44.86	44.34	10	16	8 (80.00)	8 (50.00)	2 (20.00)	8 (50.00)
Chr 42	46.05	43.60	9	11	9 (100.00)	9 (81.82)	0 (0.00)	2 (18.18)

^a^Each element is separated into two columns, indicated as H and L, representing the data from chromosomes containing homoeologous genes with relatively high and low dGC, respectively

^b^The values in parentheses indicates percentages of homoeologous/non-homoeologous genes within each homoeologous chromosome

^c^For chromosome 10, corresponding values for upstream region (Chr10_A) and downstream region (Chr10_B) of the dGC flip over are written separately

### Abundance of tRNA sequences in each subgenome

Since each subgenome showed differential codon preferences, we were also interested in abundance of the tRNA genes encoded in each subgenome to assess whether the copy number of each tRNA correlates with the codon usage preferences of each subgenome. Using tRNA gene prediction software, 37 and 39 tRNA genes were detected in the Fso_h and Fso_l subgenomes, respectively (76 genes in total encode 34 types of tRNAs with different anti-codon). The number of predicted tRNA genes is less than those in the model diatom *T. pseudonana*, which was 131 [34]. Therefore, we might not predict a complete set of tRNA genes in *F. solaris* due to sequencing incompleteness of its draft genome. Of these 34 anti-codon types of tRNA genes, only five showed multiple copies in each subgenome; AGC (Ala), GUC (Asp), GCC (Gly), AAU (Ile) showed two copies in each subgenome, and UCA (terminal codon) showed three and two copies in Fso_h and Fso_l, respectively (Additional file 2: Table S3). According to the codon preference in each subgenome, three types of anti-codons with multiple copies; GUC, GCC, and UCA correspond to codons (GAC, GGC, and UGA, respectively) preferentially found in Fso_h, while the other two; AGC and AAU correspond to codons (GCU and AUU, respectively) that characterize Fso_l (Additional file 3: Figure S2). This result indicates that there is no significant difference in the abundance and variation of tRNA genes in each subgenome, thus they are not strongly correlated to the preferences of codons in each.

### Analysis of global homoeolog expression bias

To investigate whether homoeologous gene expression in *F. solaris* is biased towards specific subgenomes, transcription levels were compared between homoeologous gene pairs during three different time points of culturing - namely before oil accumulation started (48 h of culture), during oil accumulation (96 h of culture), and after intracellular oil levels saturated (144 h). Of the 9007 pairs of homoeologous genes, 1406 pairs showed either no or very low levels of expression (with RPKM < 1 being used as cut-off) of both homoeologous genes across all three time points. Therefore, for subsequent analyses, we explored the transcription levels of the remaining 7601 homoeologous gene pairs, which have almost identical distribution of RPKM values at each time point (Additional file 6: Figure S5A).

**Fig. 3:**
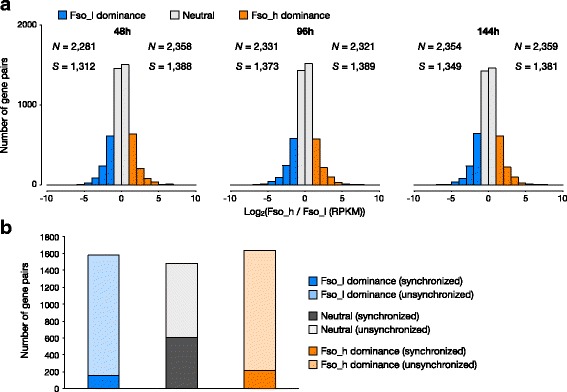
Numbers of homoeologous genes expressing with a bias towards one of the specific subgenomes. **a** Histograms of genome-wide homoeologous expression bias of homoeologous gene pairs at three different stages during oil accumulation. *N* values represent the number of genes expressing towards the subgenome A or B. *S* values indicate the number of genes counted as biased expression due to silencing of the other homoeologous pair (not included in histograms). **b** Comparison of number of synchronized and unsynchronized homoeologous gene pairs throughout 3 time points

Global expression analysis revealed that approximately 61% of homoeologous gene pairs showed biased expression towards a specific subgenome at all time points (Fig. 3a). Among these biased homoeologous pairs, half of them showed bias towards Fso_h, and the other half showed bias towards Fso_l, indicating that there is no significant global homoeolog expression bias towards a specific subgenome in *F. solaris.* However, highly expressed genes (RPKM > 50 or 100) showed biased expression towards Fso_h, with significant differences in numbers of biased homoeologous pairs (*p* < 0.05, chi-squared test) (Additional file 6: Figure S5B, C). These genes also showed greater intensity of bias, most easily observed in genes with RPKM > 500 (Additional file 6: Figure S5D). Comparison of biased homoeolgous gene expression at each time point revealed that the majority of biased genes maintained their direction of homoeolog bias throughout the three time points. Of these continuously biased genes – 1639 genes were continuously biased towards Fso_h, and 1580 genes were continuously biased towards Fso_l – we found no significant differences in functions based on analysis of GO terms (Additional file 7: Figure S6).

To investigate further, we also analysed the synchronicity of expression profiles between the pairs of homoeologous genes in each group of bias patterns, as we have studied the global expression synchronicity previously [29]. The proportion of synchronously expressed pairs in the group of consistent Fso_h-biased, consistent Fso_l biased, and Neutral (non-biased) were 12.9, 9.7 and 40.9%, respectively (Fig. 3b). These percentage distributions were significantly different from the global percentage of synchronized homoeologous gene pairs, which was 20% (*p* < 0.05, chi-squared test). These data suggest that biased genes are more likely to display unsynchronized expression profiles, while neutral homoeologous gene pairs frequently display the synchronized profiles.

To obtain further fundamental insights into the mechanisms of homoeolog expression bias, the promoter regions of homoeologous genes were also analysed. For this, 500 bp upstream of the transcription start site (TSS) was defined as promoter region, as has been done in other studies [40, 41]. Motif sequence analysis of consistently biased homoeologous gene pairs revealed several motifs which exist in specific subgenome with more significant abundancy (Additional file 2: Table S4). For instance, motif “TABASTA” (B and S are nucleotide base codes for “C or G or T” and “C or G”, respectively) is the most abundant motif we discovered in this study. This motif exists in the promoter regions of 2113 and 1588 homoeologous genes in Fso_h and Fso_l subgenomes, respectively, which include the 600 homoeologous gene pairs containing this motif in both copies of the pair. If only one gene contains the TABASTA motif within homoeologous gene pairs, genes containing this motif tend to show higher expression than the other homoeologous gene pairs (Additional file 8: Figure S7A). This indicates that this motif might act as transcription enhancer. Although the functions of these motifs remain to be elucidated, our result suggests the involvement of promoter motifs conferring homoeolog expression bias in *F. solaris*. In addition, GO analysis revealed that the TABASTA motif is significantly enriched in promoter region of genes with specific annotations (FDR < 0.05, Fisher’s exact test)(Additional file 8: Figure S7B). For example, metal ion binding transcription factor zinc finger, and stress responding genes such as heat shock proteins and E3 ubiquitin ligases contained the motif in their promoter region (Additional file 2: Table S5). These indicate that some promoters regulate the expression of genes with specific functions or pathways and cause homoeolog expression bias.

### Homoeolog expression bias in metabolic pathways

**Fig. 4:**
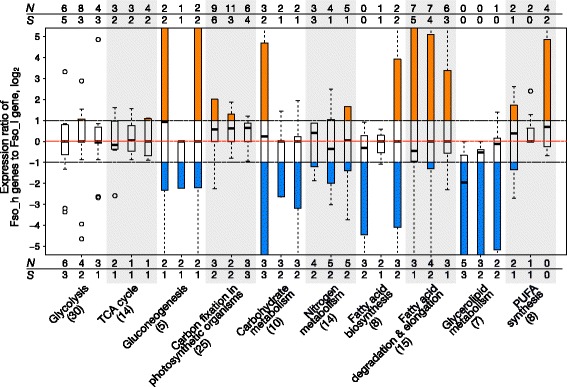
Range of homoeolog expression bias in different metabolic pathways. Box plot of log_2_ showing the expression ratio of homoeologous gene pairs in each metabolic pathway. Red dotted line indicates the equal ratio log_2_. Black dotted line indicates the threshold of expression ratio where the homoeologous gene pair is defined as biased (log_2_FC (fold change) > 1). Orange and blue colours of the boxes indicate the direction of homoeolog expression bias, towards Fso_h and Fso_l, respectively

Because *F. solaris* is known to display unique phenotypic characteristics, such as high oil accumulation and high growth rates, and because we observed subgenome-biased distribution of motifs in the promoters of genes with specific functions in the above analyses, we were interested to examine whether any association exists between homoeolog expression bias and certain metabolic modules, to investigate whether allopolyploidy may affect these pathways. In the present study, we focused on (1) carbohydrate and (2) lipid metabolic pathways, which directly link to the oil accumulation phenotype; (3) photosynthesis, which is the major source of carbon and reducing equivalent in diatoms; (4) cell cycle, especially cyclins, of which several diatom-specific types exist [42], and cyclin-dependent kinases; and (5) chromosome organization, which includes histones and histone-modifying enzymes that contribute to chromatin-level regulation of transcription. Although fairly large sets of genes involved in carbohydrate and lipid metabolism have been identified, those involved in photosynthesis, cell cycle and chromosome organization are more limited. In general, most of the metabolic modules and time points showed a median of log two-fold change in the range of − 1 to 1, indicating that no general bias in expression level towards one or the other subgenome was observed (Fig. 4, Additional file 9: Figure S8); this was consistent with the results of the genome-wide analysis (Fig. 3). However, some metabolic pathways showed significant numbers of gene pairs with homoeolog expression bias towards specific subgenome at certain time point. For instances, significant number of genes in carbon fixation at 96 h and glycerolipid metabolism at 48 h showed homoeolog expression bias towards Fso_h and Fso_l, respectively (*p* < 0.05 with Fisher’s exact test).

**Fig. 5:**
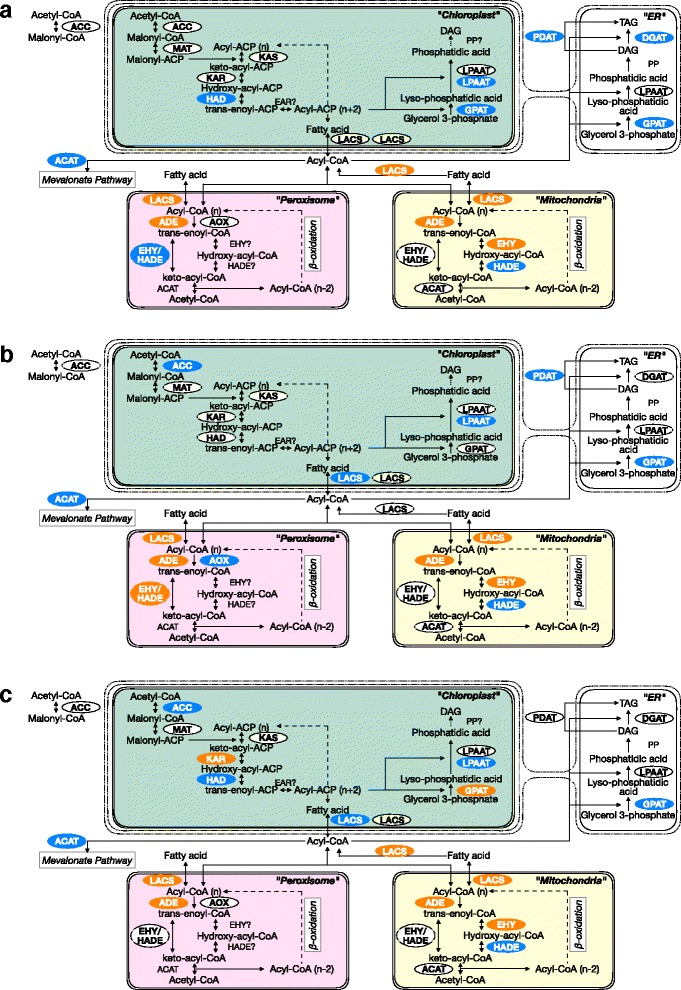
Homoeolog expression bias in fatty acid and lipid metabolism pathway. The 48 h (**a**), 96 h (**b**), and 144 h (**c**) culture. Homoeologous genes encoding enzymes that have been predicted in *F. solaris* are written inside the ellipses. The ellipses in orange and blue represent the genes with homoeolog expression bias, towards Fso_h and Fso_l, respectively

We next focused on the homoeologous gene pairs involved in lipid metabolism, such as fatty acid (FA) synthesis, FA degradation, and glycerolipid metabolism, to investigate the relationship between allopolyploidy and lipid metabolism in *F. solaris* (Fig. 5, Additional file 10: Figure S9). In general, homoeologous genes from both subgenomes were expressed and thus appear likely to contribute to lipid metabolism, which is again consistent with the results of the genome-wide analysis. However, interesting trends in bias were observed in each pathway. In the FA synthesis pathway and glycerolipid metabolism pathways which generally localised in chloroplast and endoplasmic reticulum (ER), several genes such as those coding for acetyl-CoA carboxylase (ACC, EC 6.4.1.2), β-hydroxyacyl-ACP dehydrase (HAD, EC 4.2.1.59), and lysophosphatidate acyltransferase (LPAAT, EC 2.3.1.51) localized in chloroplast; glycerol-3-phosphate (GPAT, EC 2.3.1.15) localized in ER; and phospholipid:diacylglycerol acyltransferase (PDAT, EC 2.3.1.158) showed a bias towards Fso_l at more than or equal to two time points. In particular, ACC in the chloroplast, GPAT in the ER and PDAT showed Fso_l bias at relatively high RPKM, which is in contrast to the trend observed in genome-wide analysis for high RPKM genes (Additional file 6: Figure S5B-D). In contrast, for genes encoding FA degradation and elongation processes in mitochondria and peroxisomes, more than half of the predicted homoeologous gene pairs showed a bias towards Fso_h at all three time points. Overall then, homoeolog expression bias was detected towards specific subgenomes in components of lipid metabolism occurring in specific organelles. Notably, some genes such as those encoding GPAT in the chloroplast and enoyl-CoA dehydratase/β-hydroxyacyl-CoA dehydrogenase (EHY/HADE, EC 1.1.1.35) showed bias towards both Fso_h and Fso_l in a time-point-dependent fashion. However, their expression levels were generally low, and even though homoeolog expression bias was detected, the RPKM difference was relatively small (Additional file 10: Figure S9).

## Discussion

Over 200,000 varieties of diverse and widely distributed diatoms are believed to exist worldwide [23]. This raises the question as to how diatoms acquired the plasticity that enables them to adapt to varying environmental conditions. Recently, Mock et al. reported that the cold-adapted diatom *F. cylindrus* acquired allelic variation in its diploid genome that may allow it to survive in the extreme environmental conditions of the Southern Ocean [35]. This allelic variation produces similar genomic structures in allopolyploids, resulting in variation in sequence and possibly in function of the syntenic genes. This indicates that genomic variation and its acquisition may have a considerable and important effect on diatom plasticity. The strategy by which diatoms acquire genomic variation during evolution remains unclear. We suggest that allopolyploidy is one of the possible mechanisms that confers diversified phenotypes and plasticity to diatoms.

We previously reported *F. solaris* to be the first known example of an allopolyploid microalga [29]. The ability to assign the sequenced genes to the appropriate progenitor genome is crucial to understand homoeolog expression bias, and this has been attempted in many studies of other allopolyploid organisms [6, 38]. However, a lack of genome references for closely related diatoms, such as another *Fistulifera* species which might be related to the progenitors of *F. solaris*, has thus far hampered such a classification. To our knowledge, only two strategies have been reported for classifying an allopolyploid genome into its pseudo-parental subgenomes in the absence of a progenitor genomic reference. The first method, presented by Louis et al., divided the genome of the interspecies yeast hybrid *Pichia sorbitophila* into two progenitor subgenomes P_γ_ and P_ε_ using the dGC of the homoeologous gene pairs [39]. In the case of diatom, previous study has shown that each diatom species has distinctive GC content [43], and also GC contents of diatoms even in the same genus group show distinctive difference, suggesting the distinctive nucleotide composition in each diatom species. For instances, GC contents of *Thalassiosira pseudonana* and *T. oceanica* are 46.9 and 53.4%, respectively; and GC contents of more closely related taxa to *F. solaris*, *Pseudo-nitzschia multiseries* and *P. multistriata* are 41.3 and 46.4%, respectively. In addition, we investigated the dGC of orthologs in *T. pseudonana* and *T. oceanica*, which are two different genome-read diatom species in same genus group, and discovered that they have clear difference and consistent relative values in GC contents that *T. oceanica* has higher dGC orthologs than *T. pseudonana* throughout whole genome (Additional file 11: Figure S10). These results support the adequacy of using dGC to differentiate the subgenome in allopolyploid diatom. In the previous study for allopolyploid yeast *P. sorbitophila* whose genome was read by Sanger technology [39], the relationship between dGC and global GC content was not intensively discussed. As shown in Fig. 1, dGC of *F. solaris* was not well correlated to the currently analyzed chromosomal global GC content. This might be, in part, caused by similar global GC content values in each homoeologous chromosome pair generated from the draft genome sequence of *F. solaris* (Table 1). Because, in dGC analysis, only protein-coding regions were taken into account, it can happen to show the reversed relationship between the dGC and global GC content (i.e., a low-dGC chromosome show a high-global GC content and vice versa), which is also affected by the GC contents of non-coding regions. In the future study, this relationship will be analyzed in detail with more complete genome sequence data. The second method was reported by Session et al., who used it to determine the subgenomes of the allotetraploid frog *Xenopus laevis*, by identifying dispersed relics of transposable elements distinct to each progenitor [44]; however, this strategy requires the precise identification of genomic sequences with long-read, robustly assembled, and less error-prone sequence. These strategies enable the determination of the subgenomes of allopolyploid organisms using only their genomic sequence information. In addition, although it has not been used for allopolyploid research, codon frequency bias is also known to be species-specific, and has also been applied for the classification of bacteria [45, 46] and could be used to determine the subgenomes within allopolyploid genomes. Since progenitors of allopolyploid species may have become extinct in some cases, designing such strategies for dividing an allopolyploid genome without the progenitor reference may enable the study of allopolyploids with limited genomic references. Our approach for classifying the allopolyploid subgenome used only nucleotide sequence information without requiring alignment with the sequences of related species. In this respect, our study with *F. solaris* could be an important model for the analysis of allopolyploidy using limited genomic references, as well as representing the first and only model of an allopolyploid microalga.

Because this study is the first to classify an allopolyploid microalgal genome, the suitability and reliability of the subgenome classification using dGC comparison must be verified. Codon frequency analysis revealed consistent tendencies in third-codon positions and global codon usage bias with dGC classification, although the classification remains tentative until the progenitors of *F. solaris* can be identified in future studies. Improvements in the accuracy of genomic sequencing are also vital. Indeed, because we generated the sequence of the draft genome using only de novo assembly with NGS, the possibility of misassembly exists. For example, we noticed that the position where the reversal in dGC trends occurred on chromosome 10 was the boundary of two scaffolds, scaffold00160 and scaffold00105, assembled during the chromosome sequence assembly (Additional file 1: Figure S1A). We nonetheless confirmed by PCR the scaffold assembly of the corresponding region in chromosome 10′, where the reversal in dGC trend was observed within a single scaffold (Additional file 1: Figure S1B, C). From this confirmation, the region between fso:g15942 and fso:g15955, which are the pair of homoeologous genes located at the terminal of scaffold00160 and 00105, were also examined. Primers were designed to anneal either the non-coding or non-homoeologous gene region, allowing to amplify the chromosomal 10′ specific sequence. The length of PCR products validated by electrophoresis matched the expected length from our scaffold sequence. In spite of this recombination event, it should be noted that our transcriptome analysis is not affected because we used dGC to classify the subgenome in the reversal region of chromosome 10/10′. Furthermore, since genomic context is crucial information for genome engineering, improvements in the accuracy of genomic sequencing are essential. Because our strategy for separating the subgenomes into allopolyploid genomes does not require dense sequence genomic information, our approach appears to be a reasonable option for an organism for which only a draft genome is available.

A non-homoeologous gene, located on only one of the subgenomes, could be formed in three ways: (1) it could be a unique gene inherited from a progenitor, (2) it could be a unique gene formed after interspecies hybridization by neofunctionalization - the acquisition of a new function by a copy of a homoeologous gene, or (3) the gene has undergone gene fractionation—the loss of a copy of homoeologous gene [38, 47]. Previous allopolyploidy studies have reported that gene fractionation often occurs unevenly, frequently showing the preference to one of the specific subgenomes in many plants [47] such as *Arabidopsis* [48], *Brassica* [18, 49, 50]*,* maize [51, 52], *Gossypium* [15, 53], as well as in the amphibian *X. laevis* [44]. This phenomenon results in uneven numbers of genes in each subgenome. Therefore, the unified biased pattern in the number of non-homoeologous genes in the subgenomes of *F. solaris* could be a result of such phenomena; this also supports the classification of homoeologous chromosomes to appropriate subgenomes. These data might also support the occurrence of an intergenomic rearrangement in chromosome 10 following interspecies hybridization. The exceptions in the case of chromosomes 27, 38, 41, and 42 might be owing to incomplete genome information. Chromosomes 27 and 38 are chromosomes in which telomeric repeats have not been found in either termini of the sequences [29]. In the homoeologous chromosome 27 pair, a short region with a high density of non-homoeologous genes was sequenced in chromosome 27′ alone, which may cause more non-homoeologous genes in the Fso_l chromosome. Chromosomes 41 and 42 were the two shortest chromosome pairs and might contain more genes not identified during the previous genome analysis.

Homoeolog expression bias in intracellular compartments is difficult to assess in diatoms. In general, in higher plants both mitochondria and chloroplasts are typically inherited from the maternal progenitor [54, 55]. Previous studies have reported that the homoeologous genes that encode the proteins localized in these organelles show expression bias towards the maternal subgenome [56, 57]. In diatoms, organelle inheritance during sexual reproduction differs widely between species. This results in some species showing single-progenitor inheritance like that seen in higher plants, while some other species show inheritance of the organelle from both progenitors [58–60]. Therefore, the organelle-specific homoeolog expression bias observed in *F. solaris*, the genes localised in chloroplast which involving in FA synthesis are preferentially expressed from Fso_l subgenome; and the genes localised in mitochondria and peroxisome which involving in FA degradation and elongation are preferentially expressed from Fso_h subgenome, could be considered to have originated via two patterns, with the organelle inherited from different progenitors or a novel expression pattern with organelle preference being acquired during evolution. Unlike other diatoms, which contain multiple chloroplasts [58], *F. solaris* contains only a single chloroplast [28]. Thus, it could be a useful model to analyse the inheritance of organelles during sexual reproduction in diatoms, which could facilitate further analyses of homoeolog expression bias in different intracellular compartments.

The molecular mechanism of homoeolog expression bias remains unclear. However, allopolyploidy frequently shows unique expression profiles and subgenomic preferences [10, 13] which are believed to be important for establishment of novel phenotypes distinct from those conferred by the parental genotypes. One possible cause to be considered is epigenetic modification. Epigenetic modifications, which imply phenomena such as DNA methylation and histone modifications, are known to be important for regulating gene expression and have been observed in many allopolyploids [2, 61]. Previous studies have revealed that rRNA expression in allopolyploids is regulated by DNA methylation and histone acetylation [62, 63]. Besides rRNA, several studies have shown that epigenetic modifications in allopolyploids contribute to expression divergence of homoeologous genes [64, 65]. In the present study, we found that genes involved in epigenetic regulation, including histone modifying genes express with a homoeolog expression bias towards Fso_h (Additional file 9: Figure S8). These may support the notion that homoeolog expression bias in *F. solaris* is regulated by epigenetic modifications that are mainly driven by genes encoded on the Fso_h genome. Such a notion requires further investigation. Another possible cause is difference in promoter region. Previous study in *F. cylindrus,* which has allelic divergence in portion of genome, showed that within divergent alleles, sequential identity of non-coding region is significantly lower than coding-region, which suggest that the non-coding regions also have functional diversity which could contribute to expression differences [35]. In this study, we discovered several motifs with biased distribution towards a specific subgenome, which also showed trends of being involved in homoeolog expression bias. These results suggest the relevance of promoter-level regulation in homoeolog expression bias in this allopolyploid diatom, which will motivate us to further investigate these promoter regions in future studies.

The contribution of homoeolog expression bias to the oleaginous phenotype is not readily evident. Recent reports in allopolyploid cotton seed [66] and peanut [67] have indicated that both subgenomes generally contribute to the global transcriptome in lipid metabolism, whereas several homoeologous genes exhibit an expression bias towards specific subgenomes. Similar expression patterns were observed in *F. solaris*. Generally, an orchestrated expression pattern from both subgenomes was found, while a subtle but fairly clear homoeolog expression bias was confirmed for genes involved in glycerolipid metabolism and FA degradation. This expression bias might be the result of differences in the phenotypes of the *F. solaris* progenitors because previous allopolyploid studies have shown that homoeologous genes are differentially expressed with specific subgenomic biases under certain stressful environments, depending on which of the progenitors from which the subgenomes originated was better adapted to that environment. For instance, when allopolyploid plants (*Arabidopsis kamchatica* and *Coffea arabica* L.) with one progenitor native to cold environments and the other to temperate environments were cultured at low temperatures, they preferentially expressed the stress-responding gene family from the subgenome derived from the cold-tolerant progenitor [11, 68]. These studies indicate that the subgenome derived from the progenitor more adapted to certain stresses tends to more strongly contribute to specific metabolic processes under those stresses, as with *F. solaris*, which accumulated neutral lipids under nutrient deprivation stress. Meanwhile, genes involved in glycerolipid metabolism showed homoeolog expression bias towards subgenome Fso_l. Based on previous studies of allopolyploid plants [11, 68] and the data obtained in this study, we assume that the progenitor from which Fso_l originated might preferentially accumulate neutral lipids in response to nutrient deprivation stress.

## Conclusions

In conclusion, this study presents pseudo-parental subgenome information for the first-reported allopolyploid microalga *F. solaris* and accompanying homoeologous expression patterns, focusing in particular on homoeolog expression biases in lipid metabolism. We have revealed that while both subgenomes contribute to the global transcriptome, subgenomic preferences exist in genes involved in several parts of the lipid metabolic pathway. Our results highlight the possibility that allopolyploidy contributes to the oleaginous characteristics of *F. solaris*. This novel aspect of allopolyploidy in a diatom may be advantageous for biofuel production in this microalga.

## Methods

### Genome and gene sequence data

Genome, gene, and protein sequences from *F. solaris,* which have been generated and annotated in a previous study, were used [29]. In this study, we calculated global GC content of chromosomes composed of multiple scaffolds whose sequences are available from the GenBank (Accession number: BDSP00000000, see also availability of data and material). Subsequently, each pair of 42 chromosomes were denoted as chromosome X and chromosome X’ to indicate the homoeologous chromosomes with higher GC content and lower GC content, respectively.

### Homoeologous GC content ratio calculation

dGC was calculated as described by Louis et al. [39]. The GC content percentage of coding region of all homoeologous genes along the chromosomes was computed with the R Bioconductor package Biostrings [69], and the ratio against the corresponding homoeologous chromosome of the pair was calculated for each pair using the following equation;

$$ {\mathrm{dGC}}_{\mathrm{X}}={\mathrm{GC}}_{\mathrm{X}}/\left(\left({\mathrm{GC}}_{\mathrm{X}}+{\mathrm{GC}}_{{\mathrm{X}}^{\prime }}\right)/2\right),{\mathrm{dGC}}_{{\mathrm{X}}^{\prime }}={\mathrm{GC}}_{{\mathrm{X}}^{\prime }}/\left(\left({\mathrm{GC}}_{\mathrm{X}}+{\mathrm{GC}}_{{\mathrm{X}}^{\prime }}\right)/2\right) $$

where GC_X_ and GC_X’_ in the equation indicate the GC content percentage of the homoeologous genes belonging to chromosome X (higher GC content) and chromosome X’ (lower GC content), respectively. The two dGC values for each homoeologous gene pair were plotted along each homoeologous chromosome in the order in which they appeared in the chromosome from its 5′ terminus, with a simple moving average of 11 genes [39]. In some chromosomal regions, the gene pairs were not retained at their syntenic positions due to chromosomal events such as transposition. Therefore, the basic plotting order followed the order in which the homoeologous genes appearing on chromosome X. The same calculation was performed in terms of GC content percentage specifically at the third codon positions (dGC3).

### Codon usage trend analysis

The frequencies of codons in each homoeologous genes, calculated by the R Bioconductor package Biostrings [69], were summarized into each belonging chromosome; thus, the sum of the codon frequency of the homoeologous genes in each subgenome was calculated. The calculated frequency values were then standardized, and PCA on correlation matrix was performed. Factor loadings for each codon in principal component 1 (PC1) and principal component 2 (PC2) were calculated by multiplying the eigenvector with the standard deviation of the principal component.

### RPKM calculation from RNA-seq

RNA-seq datasets from *F. solaris* cultured in f/2 media for 48, 96, and 144 h, which generated as part of our previous study [29], were used. The sequenced reads were mapped onto the reference genome using the TopHat software v2.0.14 [70], and reads per kilobase per million (RPKM) values were computed using the Genedata Expressionist software v9.1.1a (Genedata AG, Basel, Switzerland). Genes with RPKM > 1 were regarded as expressed genes [71].

### Gene annotation

The genes involved in specific metabolic pathways were investigated and annotated in three steps. Firstly, protein BLAST against *F. solaris* protein coding amino acid sequences with corresponding amino acid sequences of *P. tricornutum* and *T. pseudonana* were performed. The query sequences were collected from the KEGG pathway. Hits with e-value < 10^− 5^ were then checked for sequence domain and motif content using Interproscan [72], to verify that they contained the relevant domains as the targeted protein. Finally, phylogenetic trees of the sequences with the relevant domains and the query were drawn by CLUSTALW with unweighted pair group method with arithmetic mean (UPGMA) [73]. The sequence was identified and annotated with the protein function if it located in the same cluster as the annotated query sequence. Gene ontologies (GO) were annotated by Blast2GO software [74]. tRNA genes were identified with softwares tRNAscan-SE [75] and ARAGORN [76].

### Evaluation of bias within homoeologous gene expression

We defined genes with biased expression as either those homoeologous genes, with higher RPKM values two-fold or greater than the paired gene, as in previous studies [20], or those with no expression from one of the homoeologous genes. When assessing homoeolog expression bias in metabolic pathways and in order to compare only the functionally similar homoeologous gene pairs, homoeologous gene pairs that localised in different intracellular compartments were excluded. Pearson’s chi-square test or Fisher’s exact test were performed for each sample, depends on the sample size.

### Promoter analysis

Sequences 500 bp upstream of the TSS of each gene were extracted from the genome and used as the promoter sequences. TSS of each annotated gene has been predicted by gene prediction software in previous study [29]. For motif analysis, tools of MEME Suite (v.4.12.0) were used with default settings [77]. Motif discovery was performed with Discriminative Regular Expression Motif Elicitation (DREME) with entire homoeologous gene promoters and extracted subgenome independent promoters. To evaluate the biased distribution, Analysis of Motif Enrichment (AME) was used. Functional annotation was performed with Motif Comparison Tool Tomtom against databases of transcription factor binding sites, JASPAR Core 2016 Plants [78] and Plant cistrome database [79].

## Additional files

Additional file 1: **Figure S1.** Structure of dGC flip over region on chromosome 10. **(A)** Orange and blue colors of the pentagon arrows represent the different dGC values in corresponding homoeologous genes; orange indicates the genes with relatively higher dGC and blue indicates the genes with relatively lower dGC within the pair. Each homoeologous gene pair are connected with dotted lines. The horizontal dotted line at the ends of some of the scaffolds indicates that the scaffold continues beyond what is shown in the figures. The gene map is not drawn to scale. **(B)** Scaffold assembly of dGC flip over region in chromosome 10′, which is rounded by red line in (A), was validated by PCR. The schematic figure represents the 8 amplified regions in PCR experiment. Vertically printed numbers indicate the base position within the scaffold. Each amplified region has overlapped region to the next. Primers for this experiment was designed to annealed on either non-coding region or non-homoeologous gene region, to avoid the non-specific amplification of paired homoeologous gene. **(C)** Length of PCR products were analyzed by electrophoresis through a 0.8% (wt/vol) agarose gel. Indicated lane numbers are corresponding to the amplified region shown in (B). (PDF 69 kb)

Additional file 2: **Table S1.** Codon usage in genome and subgenome of *F. solaris*. Codon usage in (**A**) genome and (**B**) subgenome of *F. solaris* in per mil. (A) The table includes the codon usage of all genes predicted in *F. solaris*, including homoeologous genes, non-homoeologous genes, and genes predicted on scaffolds not assembled on chromosomes. (B) The table includes the codon usage of homoeologous genes and non-homoeologous genes in each subgenome. The values corresponding to Fso_h and Fso_l were written in left (bold) and right (italic) of each column, respectively. Values in parentheses represent the ratio of codon usage between Fso_h and Fso_l (Fso_h / Fso_l); **Table S2.** Top 10 and bottom 10 loadings of PC1 and PC2 from the PCA based on codon usage frequency; **Table S3.** tRNA genes discovered in each subgenome of *F. solaris*; **Table S4.** Nucleotides sequence motifs discovered in promoter region of consistently biased homoeologous genes in *F. solaris*; **Table S5.** Lists of genes which contained the motif TABASTA in promoter region. (XLSX 183 kb)

Additional file 3: **Figure S2.** Codon usage frequency of subgenomes Fso_h and Fso_l. (**A**) Codon usage frequency of each of the subgenomes are compared with genomes of the model diatoms *P. tricornutum* and *T. pseudonana*. Codon usage of *P. tricornutum* and *T. pseudonana* were calculated from the nucleotide FASTA file of the newest version of filtered gene models (v2.0 for *P. tricornutum* (Phatr2) and v3.0 for *T. pseudonana* (Thaps3)) downloaded from the genome portal of the Department of Energy Joint Genome Institute (JGI) [80]. (**B**) Codon usage frequency of the subgenomes ordered in coding amino acids and (**C**) ratio of usage between Fso_h and Fso_l. Dots show the ratio of codon usage (right y-axis). Red line indicates the equal ratio, therefore the codons with the usage ratio above the red line are more frequently used in Fso_h subgenome, whereas codons with the usage ratio beneath the red line are more frequently used in Fso_l subgenome. (PDF 110 kb)

Additional file 4: **Figure S3.** Comparison of GC content at the third codon position (dGC3) between pairs of homoeologous genes in each homoeologous chromosome. The vertical axis represents the dGC3 value of each homoeologous gene, and horizontal axis indicates the relative number of homoeologous genes on each chromosome. The dGC3 value indicates the ratio of GC content at the third codon position in each homoeologous gene with respect to its corresponding pair, so if the dGC3 value is over 1 (above the horizontal axis), its GC content is higher than the other pair. Red and green lines correspond to the global GC content of chromosome; red lines indicate the dGC3 value of homoeologous gene along the homoeologous chromosome with higher global GC content and green lines indicate the dGC3 value of the gene along the homoeologous chromosome with lower global GC content. The profile of dGC3 showed perfect consistency with dGC (Fig. 1) and approximately twice more difference than dGC. We also calculated the GC content ratio of first and second codon positions; however consistency to dGC was low. This suggests that the differences in dGC of the two subgenomes was mainly derived from the GC content ratio of the third codon position. (PDF 137 kb)

Additional file 5: **Figure S4.** PCA based on codon usage frequency of dGC flip over region on chromosome 10. PCA based on codon usage frequency of dGC flip over region on chromosome 10. 10_Fso_h and _l represents the PCA result of all homoeologous genes on chromosome 10 with our tentative classification described in the Results section. These values are identical to “10” represented in Fig. 2. 10_A and 10_B represents the PCA results of chromosome 10 (high global GC content), _A includes the homoeologous genes located in the upstream region of dGC flip and _B includes the homoeologous genes located downstream. 10′_A and 10’_B represents the PCA result of corresponding region in chromosome 10′ (low global GC content). (PDF 37 kb)

Additional file 6: **Figure S5.** Homoeolog expression bias in highly expressing genes. (**A**) Box plot showing RPKM values of homoeologous genes at each time point. The minimum value of outlier (represented as a circle in the figure) is approximately 50 at every time point. (**B-D**) Histograms show number of homoeologous genes expressing in a biased fashion towards a specific subgenome. Each figure includes the homoeologous genes expressing within a certain range of RPKM values; (B) RPKM > 50, (C) > 100 and (D) > 500. *N* values in figure represent the number of genes expressing preferentially in subgenome A or B. *S* values in the figure indicate the number of genes counted as biased expression due to silencing of the other homoeologous pair (not included in histograms). (PDF 97 kb)

Additional file 7: **Figure S6.** GO terms of consistently biased homoeologous genes. Figure shows the frequency of GO terms, annotated to the second depth, within the group of homoeologous genes which were consistently biased towards Fso_h or Fso_l (corresponding to the genes in the histogram of Fig. 3b). Orange and blue colors represent the direction of homoeolog expression bias of the group, Fso_h and Fso_l, respectively. Dots in the figure represent the log ratio of frequency of corresponding GO terms between Fso_h-biased group and Fso_l-biased group. (PDF 279 kb)

Additional file 8: **Figure S7.** Expression and functional differences depending on existence of motif TABASTA in promoter region. **(A)** Figure shows the mean number of homoeologous gene pairs which showing homoeolog expression bias towards Fso_h or Fso_l (*n* = 3, ±S.D.). Bars were separated into three groups depend on whether the motif exist in promoter region of both gene of the pair, only Fso_h gene of the pair or only Fso_l gene of the pair. Asterisk show significant differences (*p* < 0.05, Welch’s test). **(B)** Figure shows the GO terms which were substantially over represented in the group of genes which contain the motif TABASTA in promoter region (FDR < 0.05, Fisher’s exact test). The vertical axis represents the relative abundance of genes which have been annotated with corresponding GO terms. (PDF 265 kb)

Additional file 9: **Figure S8.** Range of homoeolog expression bias in metabolic pathways. Box plot of log_2_ showing the expression ratio of homoeologous gene pairs in each metabolic pathway. Red dotted line indicates the equal ratio log_2_. Black dotted line indicates the threshold of expression ratio where the homoeologous gene pair is defined as biased (log_2_FC > 1). The orange and blue colors of the box indicate the direction of homoeolog expression bias, towards Fso_h and Fso_l, respectively. (PDF 37 kb)

Additional file 10: **Figure S9.** Expression profile of homoeologous genes involved in lipid metabolism at the 48, 96, and 144 h time points. Orange and blue lines represent the subgenomes of each homoeologous gene belonging to Fso_h and Fso_l, respectively. (PDF 151 kb)

Additional file 11: **Figure S10.** Comparison of GC content ratio between orthologous pair of *Thalassiosira oceanica* and *T. pseudonana*. 6463 orthologs of *T. oceanica* and *T. pseudonana* were discovered by sequence homology analysis and GC content ratio (dGC) were calculated. Vertical axis represents dGC value of each orthologs. The dGC values of orthologs along the *T. oceanica* and *T. pseudonana* were indicated as red and green line, respectively. The dGC plots for individual chromosomes were not prepared because the genome sequence of *T. oceanica* was not assembled into each chromosome. (PDF 151 kb)

## Abbreviations

ACC: Acetyle-CoA carboxylase
BLAST: Basic local alignment search tool
dGC: Guanine-Cytosine content ratio
EHY: Enoyl-CoA dehydratase
ER: Endoplasmic reticulum
FA: Fatty acid
GC: Guanine-Cytosine
GO: Gene ontology
GPAT: Glycerol-3-phosphate
HAD: Hydroxyacyl-ACP dehydrase
HADE: β-hydroxyacyl-CoA dehydrogenase
KEGG: Kyoto encyclopedia of genes and genomes
LPAAT: Lysophosphatidate acyltransferase
PC: Principal component
PCA: Principal component analysis
PCR: Polymerase chain reaction
PDAT: Phospholipid:diacylglycerol acyltranseferase
RPKM: Reads per kilobase per million mapped reads
TSS: Transcription starting site
UPGMA: Unweighted pair group method with arithmetic mean
