# Supplementary figures and images for: Homoeolog expression bias in allopolyploid oleaginous marine diatom Fistulifera solaris
Source: BMC Genomics. 2018 May 4;19:330. doi: 10.1186/s12864-018-4691-0 (PMC5935921; doi:10.1186/s12864-018-4691-0)

**A**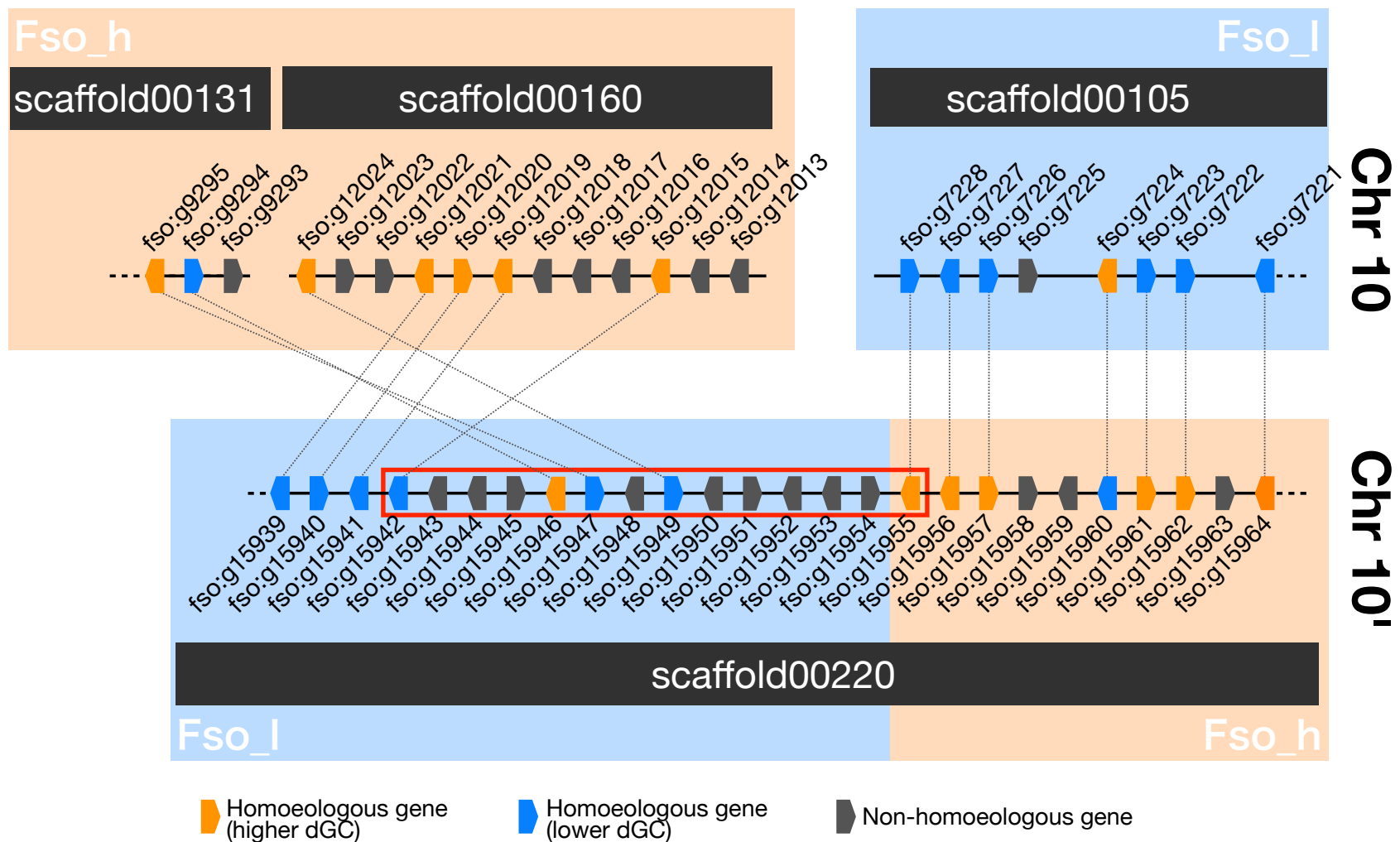**B**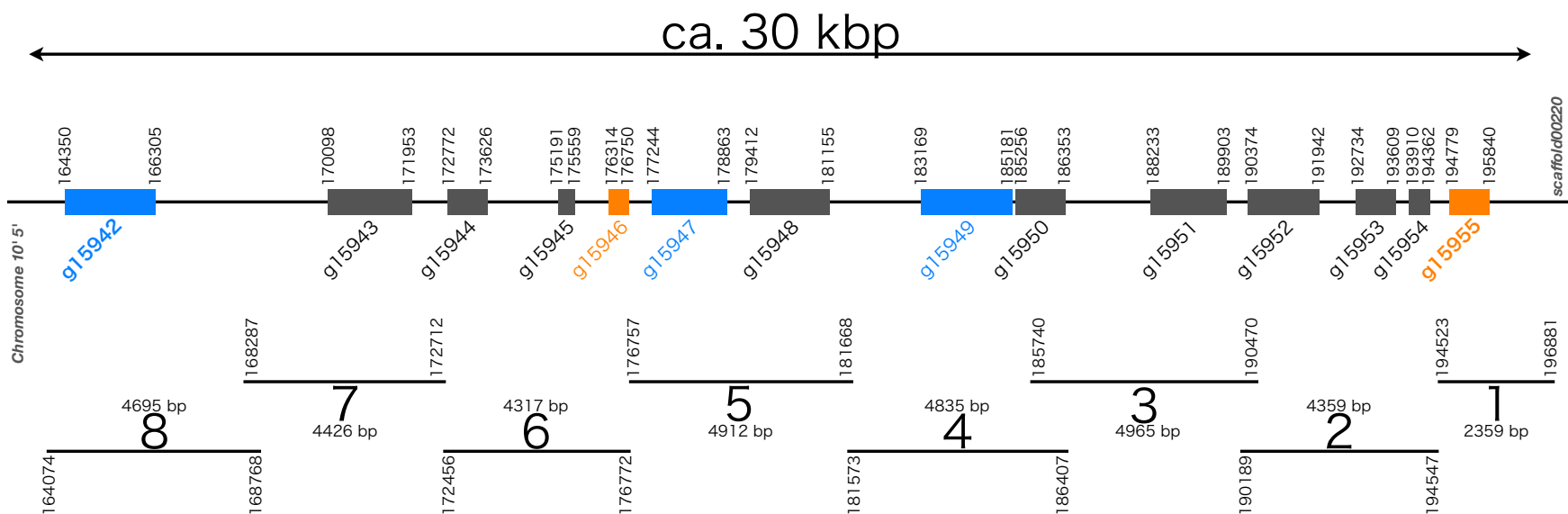**C**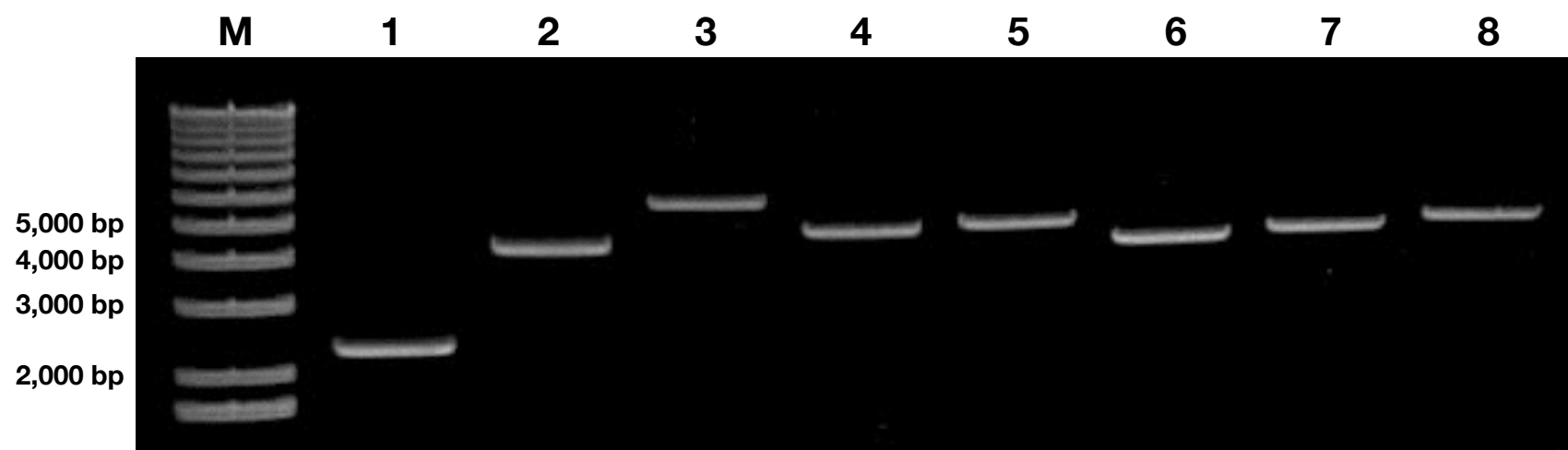

Supplement: Supplementary file 1 — Figure S1. Structure of dGC flip over region on chromosome 10. (A) Orange and blue colors of the pentagon arrows represent the different dGC values in corresponding homoeologous genes; orange indicates the genes with relatively higher dGC and blue indicates the genes with relatively lower dGC within the pair. Each homoeologous gene pair are connected with dotted lines. The horizontal dotted line at the ends of some of the scaffolds indicates that the scaffold continues beyond what is shown in the figures. The gene map is not drawn to scale. (B) Scaffold assembly of dGC flip over region in chromosome 10′, which is rounded by red line in (A), was validated by PCR. The schematic figure represents the 8 amplified regions in PCR experiment. Vertically printed numbers indicate the base position within the scaffold. Each amplified region has overlapped region to the next. Primers for this experiment was designed to annealed on either non-coding region or non-homoeologous gene region, to avoid the non-specific amplification of paired homoeologous gene. (C) Length of PCR products were analyzed by electrophoresis through a 0.8% (wt/vol) agarose gel. Indicated lane numbers are corresponding to the amplified region shown in (B). (PDF 69 kb) [file 12864_2018_4691_MOESM1_ESM.pdf]

**A**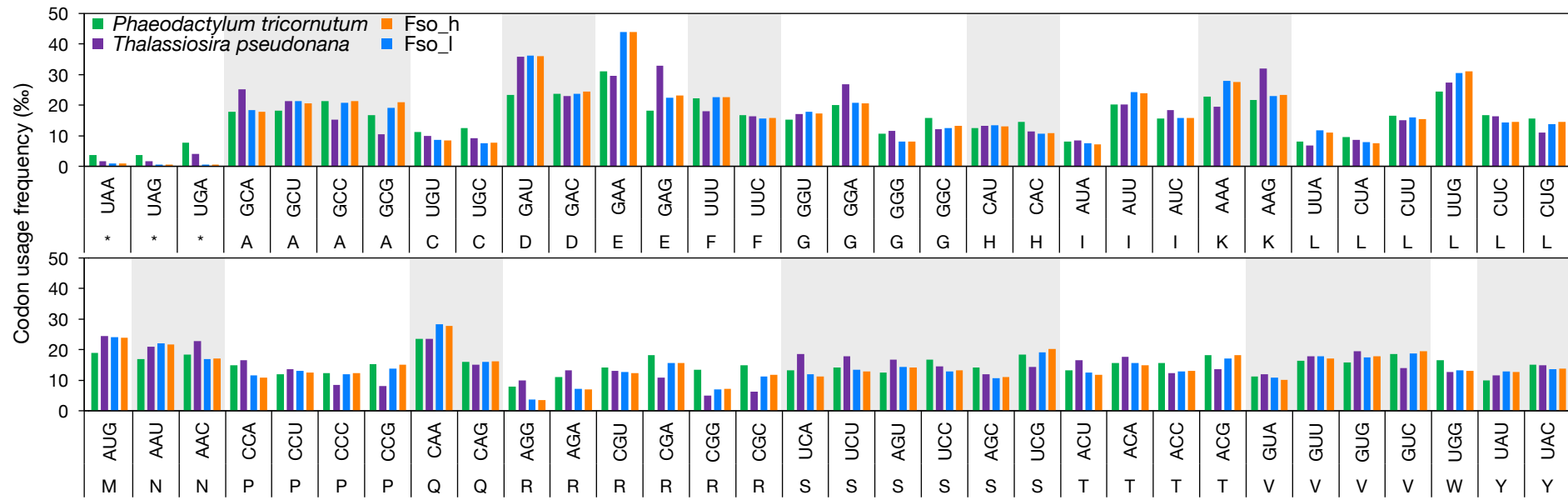**B**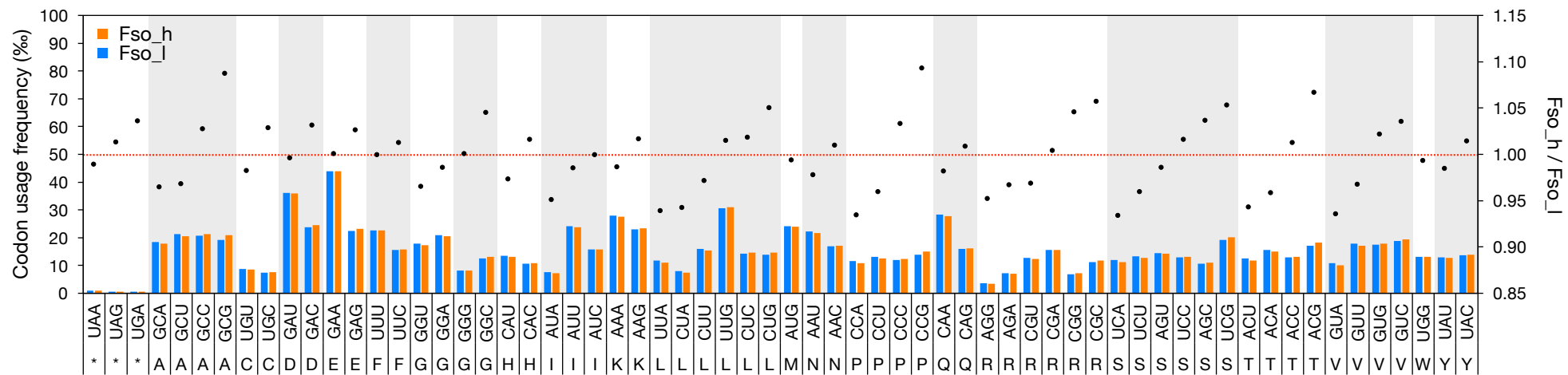**C**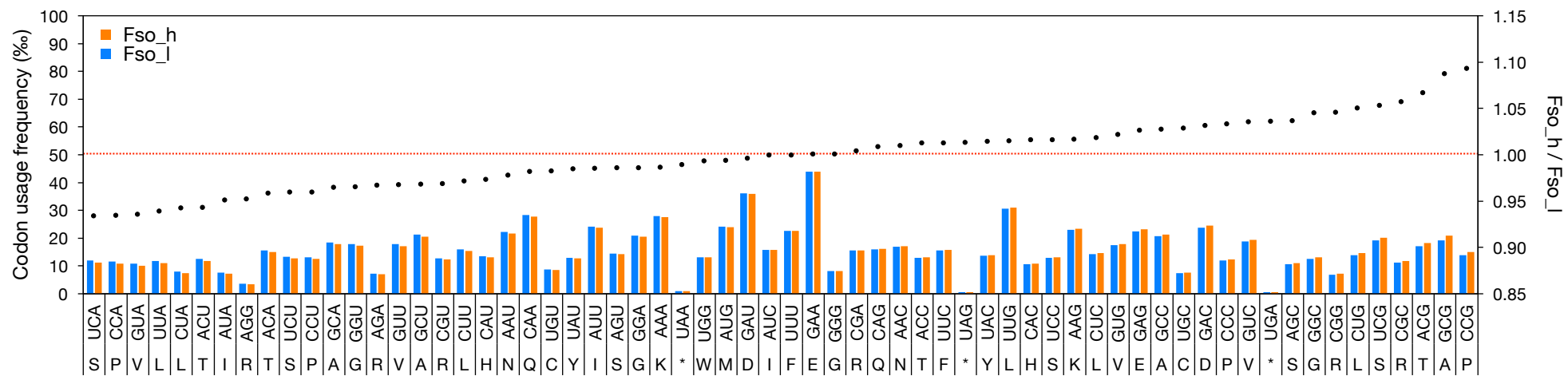

Supplement: Supplementary file 3 — Figure S2. Codon usage frequency of subgenomes Fso_h and Fso_l. (A) Codon usage frequency of each of the subgenomes are compared with genomes of the model diatoms P. tricornutum and T. pseudonana. Codon usage of P. tricornutum and T. pseudonana were calculated from the nucleotide FASTA file of the newest version of filtered gene models (v2.0 for P. tricornutum (Phatr2) and v3.0 for T. pseudonana (Thaps3)) downloaded from the genome portal of the Department of Energy Joint Genome Institute (JGI) [80]. (B) Codon usage frequency of the subgenomes ordered in coding amino acids and (C) ratio of usage between Fso_h and Fso_l. Dots show the ratio of codon usage (right y-axis). Red line indicates the equal ratio, therefore the codons with the usage ratio above the red line are more frequently used in Fso_h subgenome, whereas codons with the usage ratio beneath the red line are more frequently used in Fso_l subgenome. (PDF 110 kb) [file 12864_2018_4691_MOESM3_ESM.pdf]

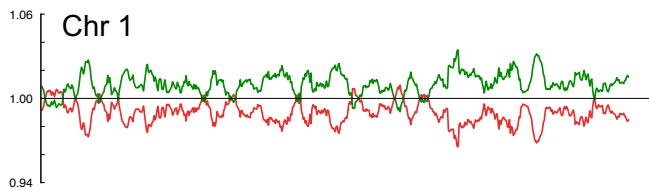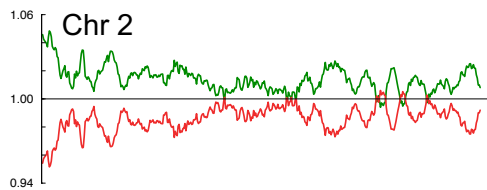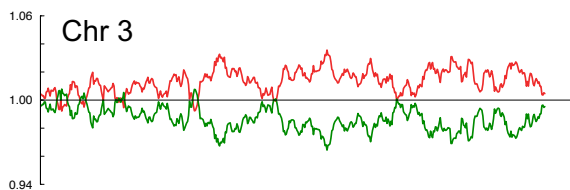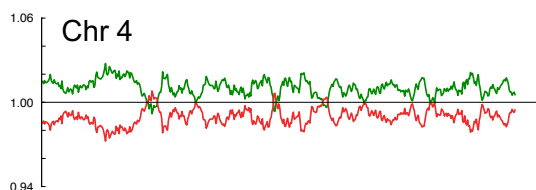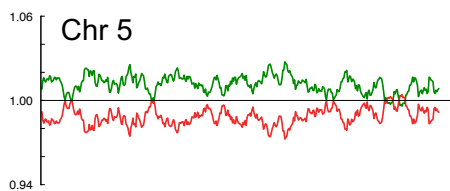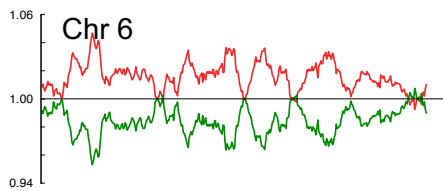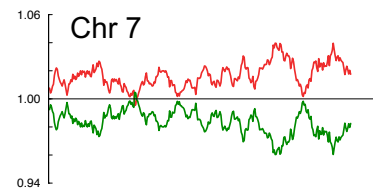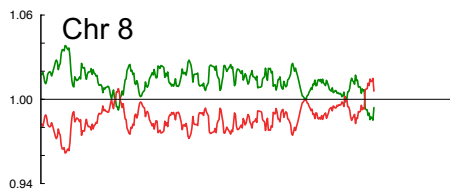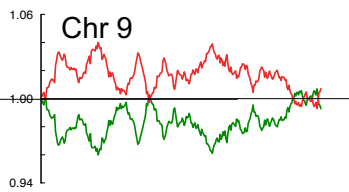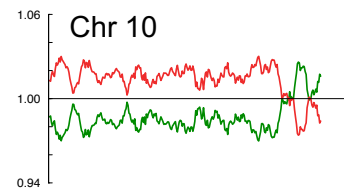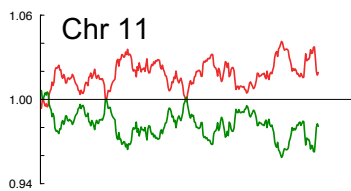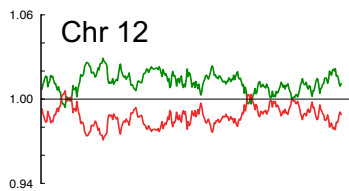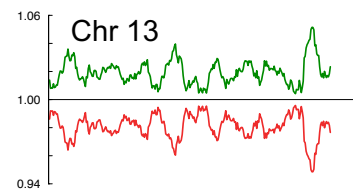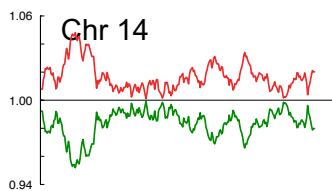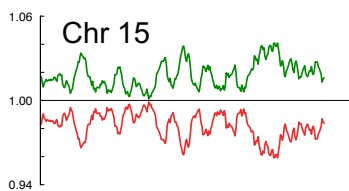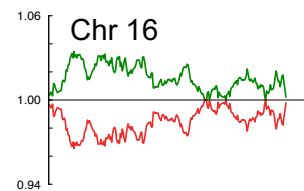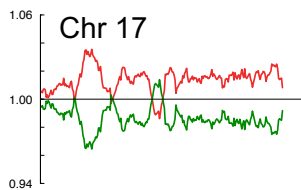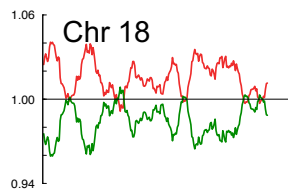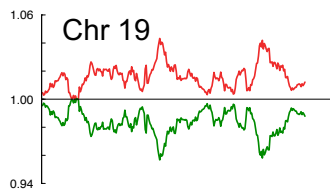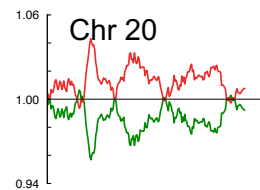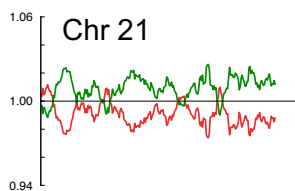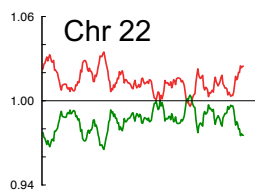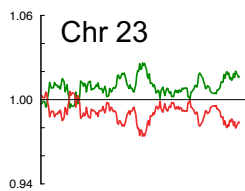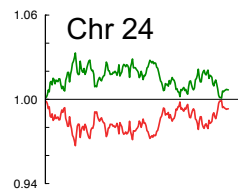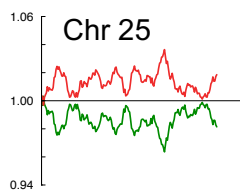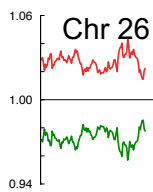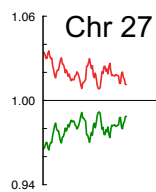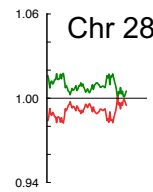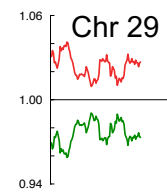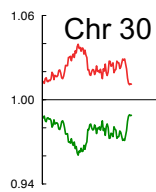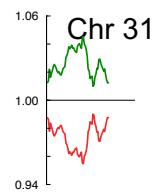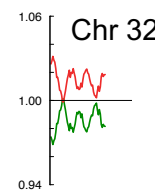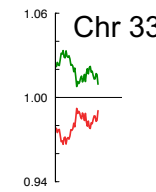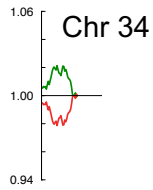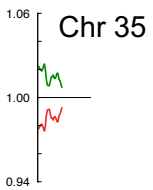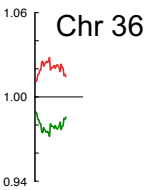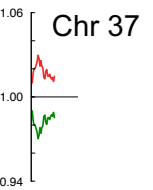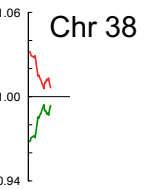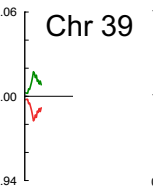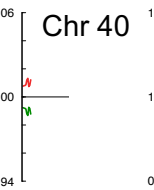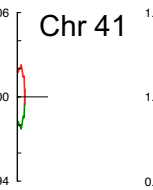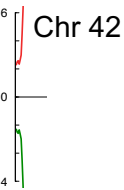

Supplement: Supplementary file 4 — Figure S3. Comparison of GC content at the third codon position (dGC3) between pairs of homoeologous genes in each homoeologous chromosome. The vertical axis represents the dGC3 value of each homoeologous gene, and horizontal axis indicates the relative number of homoeologous genes on each chromosome. The dGC3 value indicates the ratio of GC content at the third codon position in each homoeologous gene with respect to its corresponding pair, so if the dGC3 value is over 1 (above the horizontal axis), its GC content is higher than the other pair. Red and green lines correspond to the global GC content of chromosome; red lines indicate the dGC3 value of homoeologous gene along the homoeologous chromosome with higher global GC content and green lines indicate the dGC3 value of the gene along the homoeologous chromosome with lower global GC content. The profile of dGC3 showed perfect consistency with dGC (Fig. 1) and approximately twice more difference than dGC. We also calculated the GC content ratio of first and second codon positions; however consistency to dGC was low. This suggests that the differences in dGC of the two subgenomes was mainly derived from the GC content ratio of the third codon position. (PDF 137 kb) [file 12864_2018_4691_MOESM4_ESM.pdf]

# Chr10

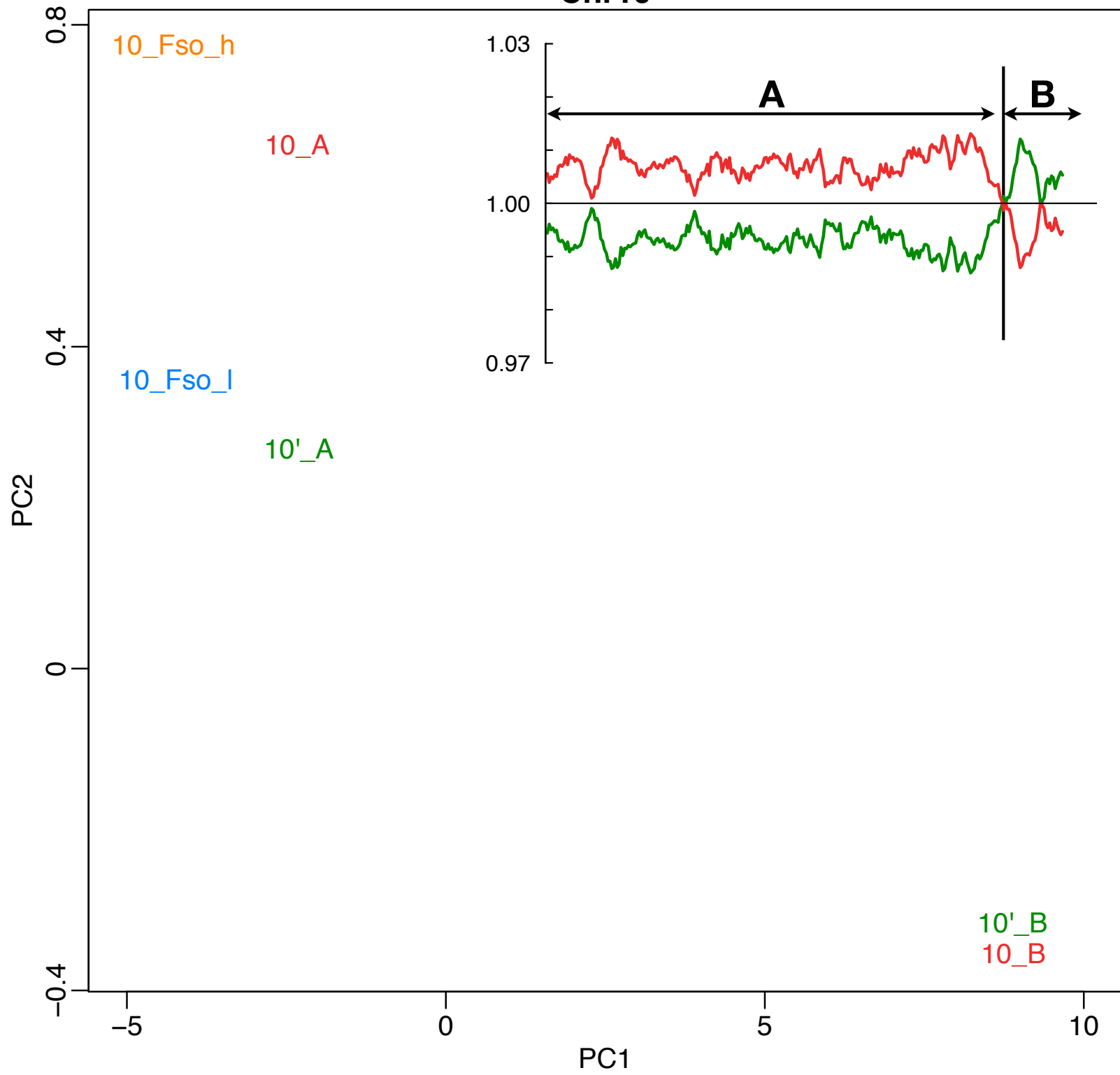

Supplement: Supplementary file 5 — Figure S4. PCA based on codon usage frequency of dGC flip over region on chromosome 10. PCA based on codon usage frequency of dGC flip over region on chromosome 10. 10_Fso_h and _l represents the PCA result of all homoeologous genes on chromosome 10 with our tentative classification described in the Results section. These values are identical to “10” represented in Fig. 2. 10_A and 10_B represents the PCA results of chromosome 10 (high global GC content), _A includes the homoeologous genes located in the upstream region of dGC flip and _B includes the homoeologous genes located downstream. 10′_A and 10’_B represents the PCA result of corresponding region in chromosome 10′ (low global GC content). (PDF 37 kb) [file 12864_2018_4691_MOESM5_ESM.pdf]

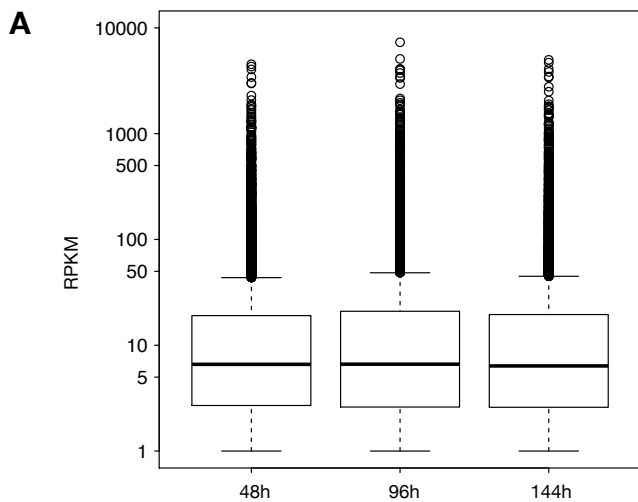

■ Fso\_h dominance
 ■ Fso\_l dominance
 ■ Neutral

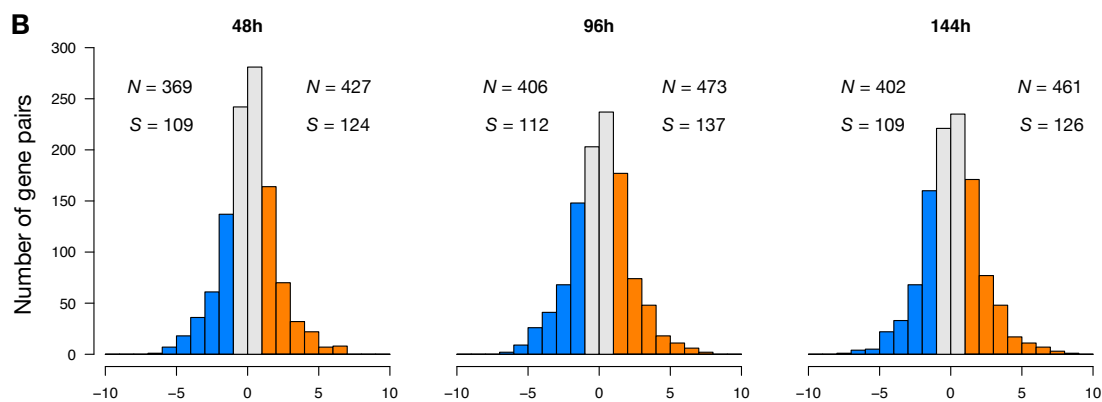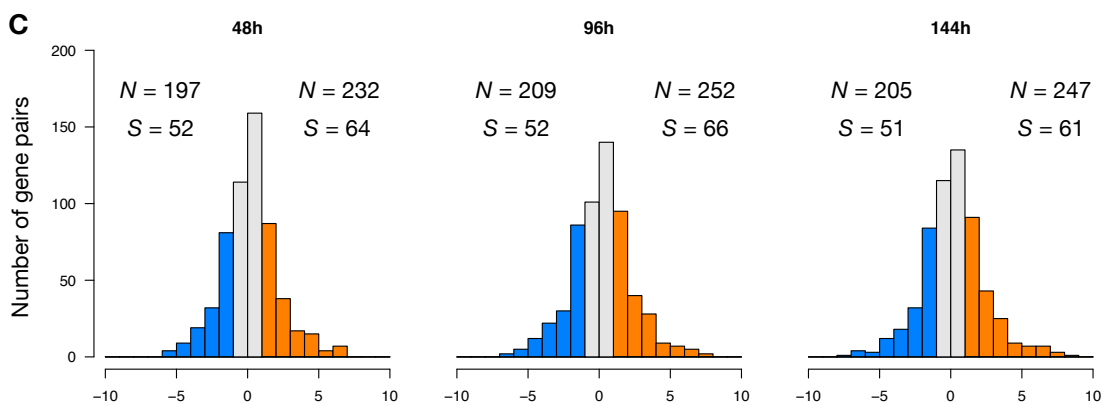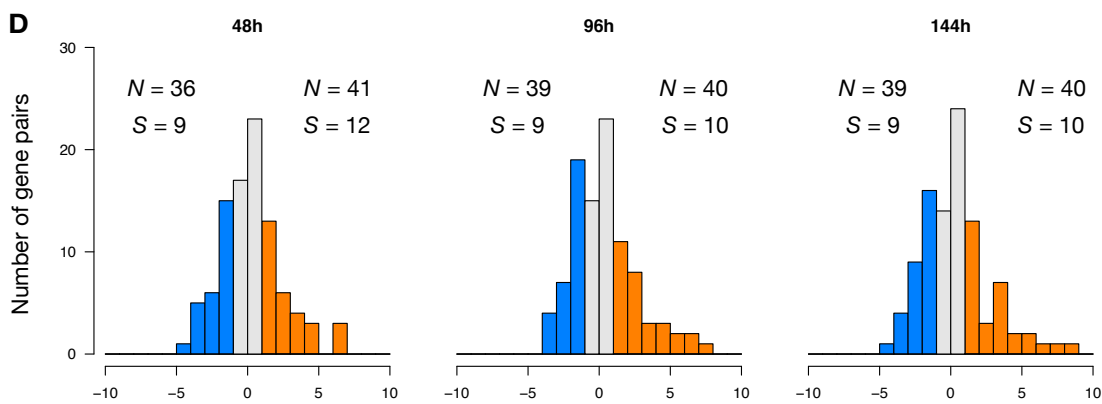

$\text{Log}_2(\text{Fso}_h / \text{Fso}_l \text{ (RPKM)})$

Supplement: Supplementary file 6 — Figure S5. Homoeolog expression bias in highly expressing genes. (A) Box plot showing RPKM values of homoeologous genes at each time point. The minimum value of outlier (represented as a circle in the figure) is approximately 50 at every time point. (B-D) Histograms show number of homoeologous genes expressing in a biased fashion towards a specific subgenome. Each figure includes the homoeologous genes expressing within a certain range of RPKM values; (B) RPKM > 50, (C) > 100 and (D) > 500. N values in figure represent the number of genes expressing preferentially in subgenome A or B. S values in the figure indicate the number of genes counted as biased expression due to silencing of the other homoeologous pair (not included in histograms). (PDF 97 kb) [file 12864_2018_4691_MOESM6_ESM.pdf]

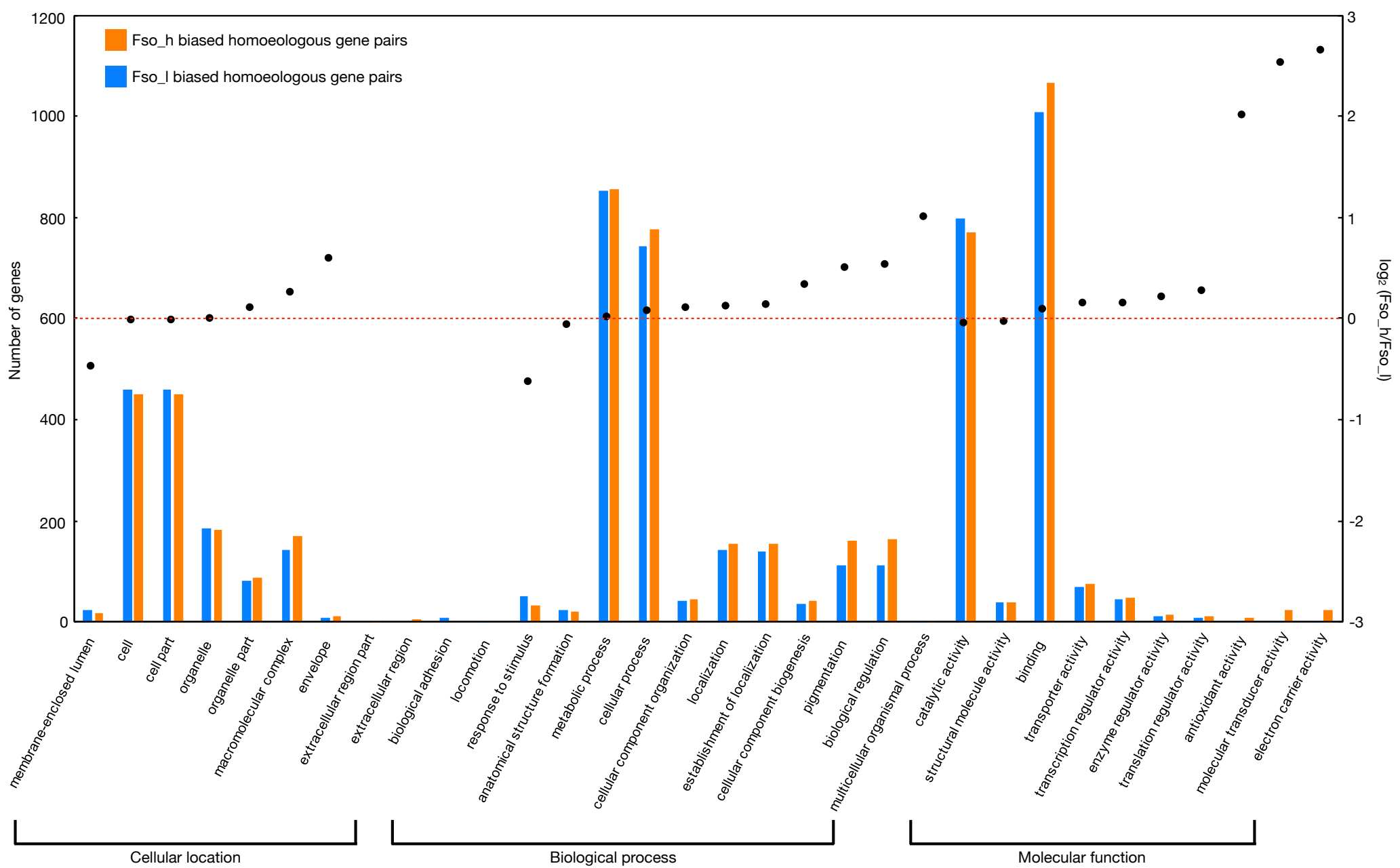

Supplement: Supplementary file 7 — Figure S6. GO terms of consistently biased homoeologous genes. Figure shows the frequency of GO terms, annotated to the second depth, within the group of homoeologous genes which were consistently biased towards Fso_h or Fso_l (corresponding to the genes in the histogram of Fig. 3b). Orange and blue colors represent the direction of homoeolog expression bias of the group, Fso_h and Fso_l, respectively. Dots in the figure represent the log ratio of frequency of corresponding GO terms between Fso_h-biased group and Fso_l-biased group. (PDF 279 kb) [file 12864_2018_4691_MOESM7_ESM.pdf]

**A**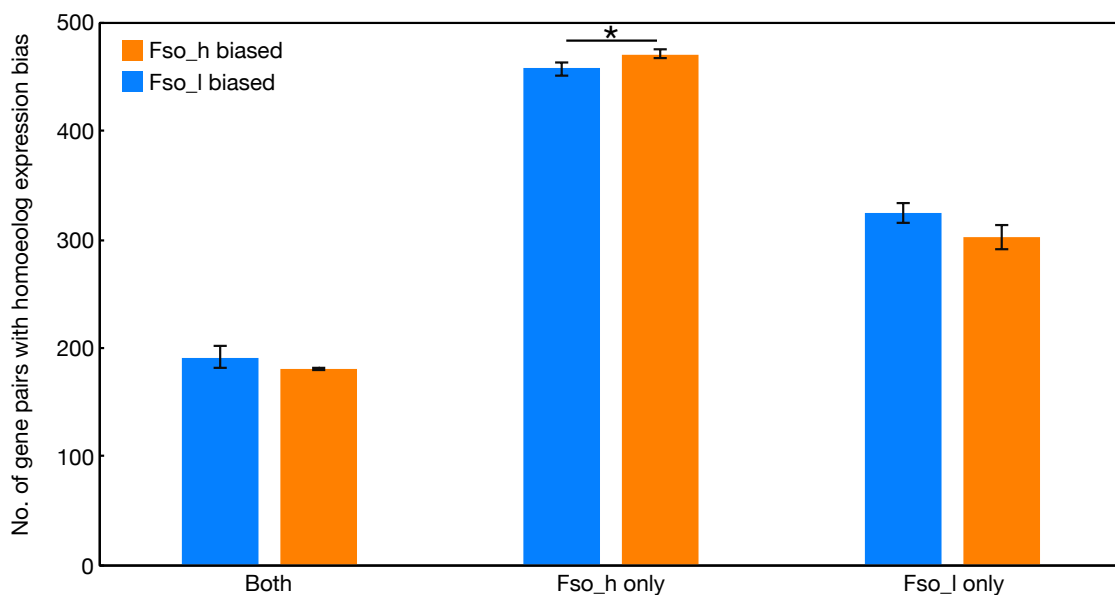**B**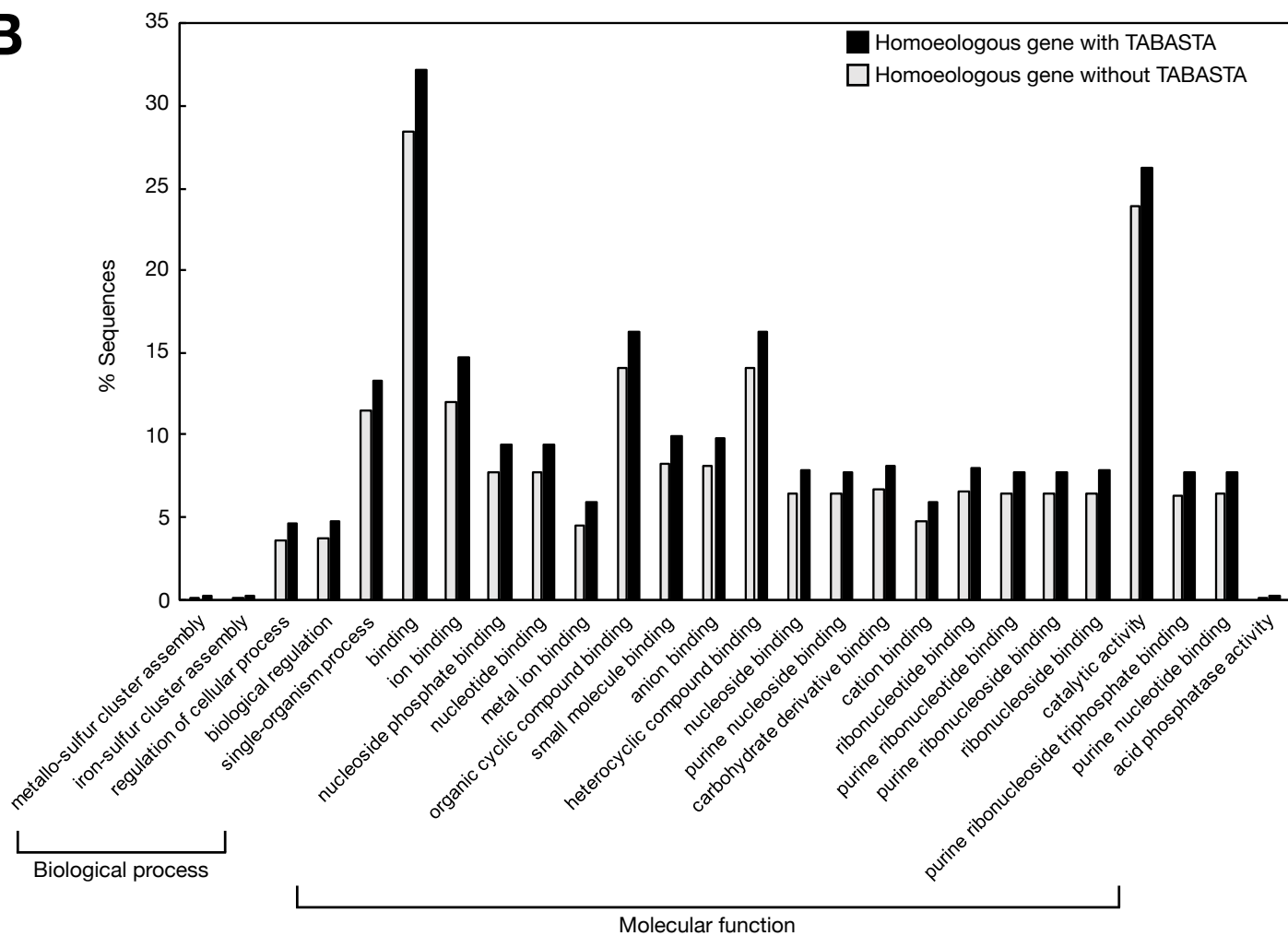

Supplement: Supplementary file 8 — Figure S7. Expression and functional differences depending on existence of motif TABASTA in promoter region. (A) Figure shows the mean number of homoeologous gene pairs which showing homoeolog expression bias towards Fso_h or Fso_l (n = 3, ±S.D.). Bars were separated into three groups depend on whether the motif exist in promoter region of both gene of the pair, only Fso_h gene of the pair or only Fso_l gene of the pair. Asterisk show significant differences (p < 0.05, Welch’s test). (B) Figure shows the GO terms which were substantially over represented in the group of genes which contain the motif TABASTA in promoter region (FDR < 0.05, Fisher’s exact test). The vertical axis represents the relative abundance of genes which have been annotated with corresponding GO terms. (PDF 265 kb) [file 12864_2018_4691_MOESM8_ESM.pdf]

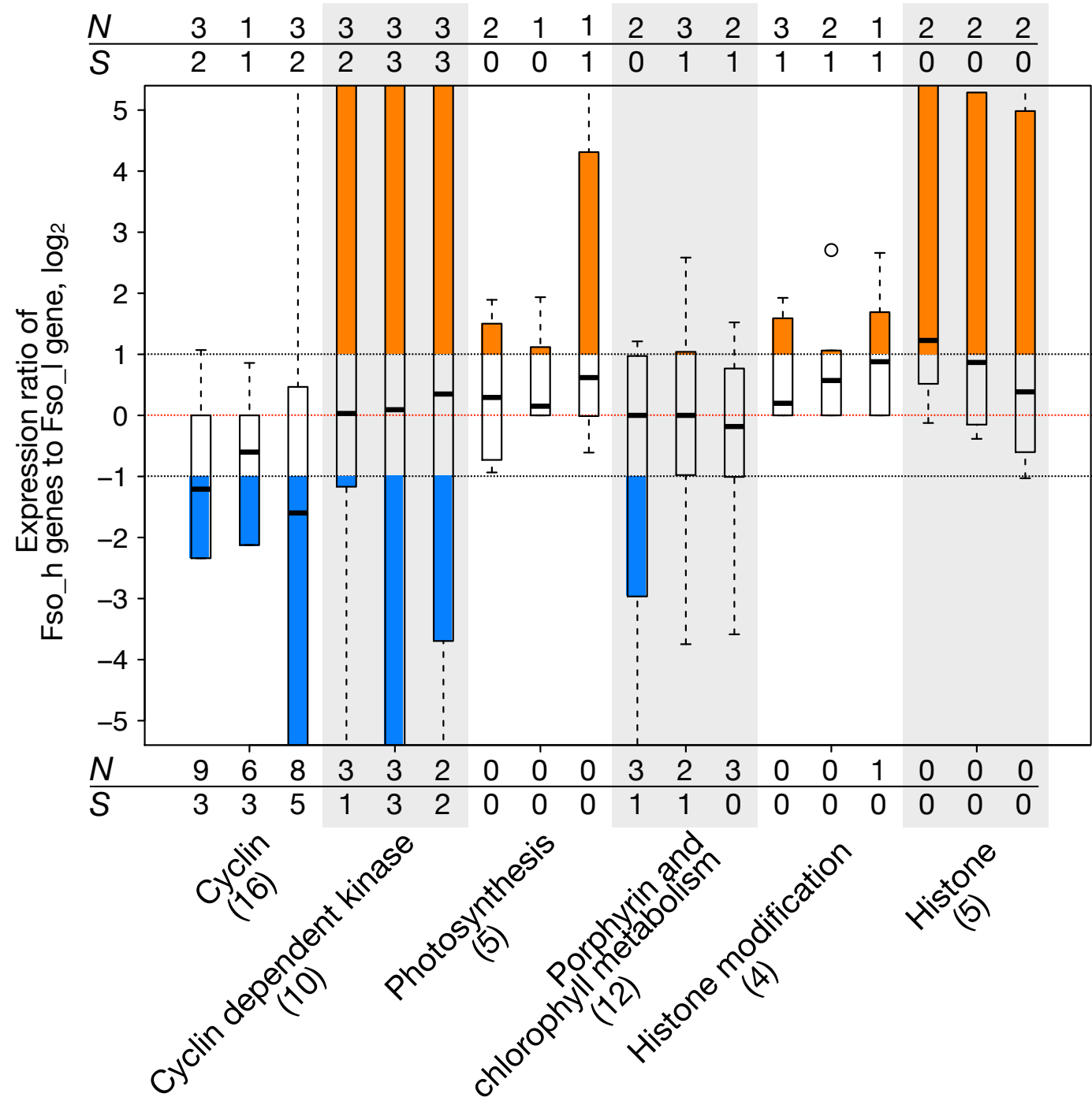

Supplement: Supplementary file 9 — Figure S8. Range of homoeolog expression bias in metabolic pathways. Box plot of log2 showing the expression ratio of homoeologous gene pairs in each metabolic pathway. Red dotted line indicates the equal ratio log2. Black dotted line indicates the threshold of expression ratio where the homoeologous gene pair is defined as biased (log2FC > 1). The orange and blue colors of the box indicate the direction of homoeolog expression bias, towards Fso_h and Fso_l, respectively. (PDF 37 kb) [file 12864_2018_4691_MOESM9_ESM.pdf]

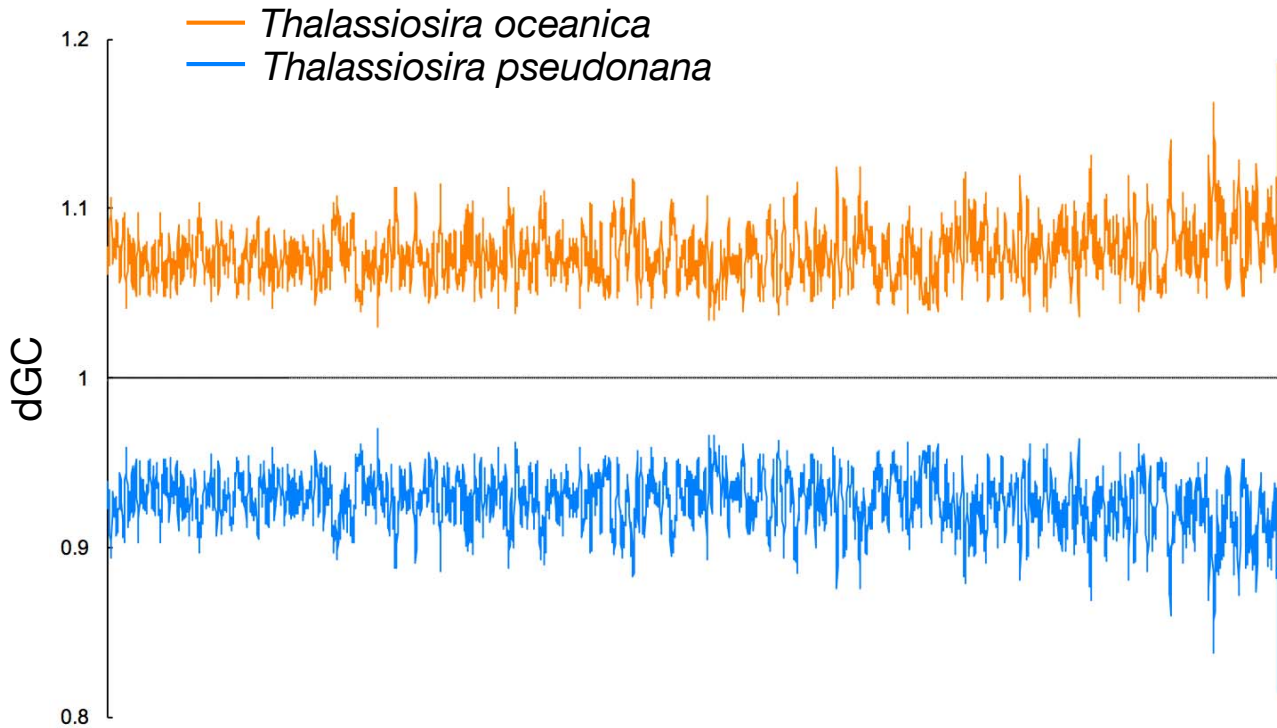

Supplement: Supplementary file 10 — Figure S9. Expression profile of homoeologous genes involved in lipid metabolism at the 48, 96, and 144 h time points. Orange and blue lines represent the subgenomes of each homoeologous gene belonging to Fso_h and Fso_l, respectively. (PDF 151 kb) [file 12864_2018_4691_MOESM10_ESM.pdf]
