# Supplementary material for: Homoeolog expression bias in allopolyploid oleaginous marine diatom Fistulifera solaris
Source: BMC Genomics. 2018 May 4;19:330. doi: 10.1186/s12864-018-4691-0 (PMC5935921; doi:10.1186/s12864-018-4691-0)
Supplement: Supplementary file 11 — Figure S10. Comparison of GC content ratio between orthologous pair of Thalassiosira oceanica and T. pseudonana. 6463 orthologs of T. oceanica and T. pseudonana were discovered by sequence homology analysis and GC content ratio (dGC) were calculated. Vertical axis represents dGC value of each orthologs. The dGC values of orthologs along the T. oceanica and T. pseudonana were indicated as red and green line, respectively. The dGC plots for individual chromosomes were not prepared because the genome sequence of T. oceanica was not assembled into each chromosome. (PDF 151 kb) [file 12864_2018_4691_MOESM11_ESM.pdf]

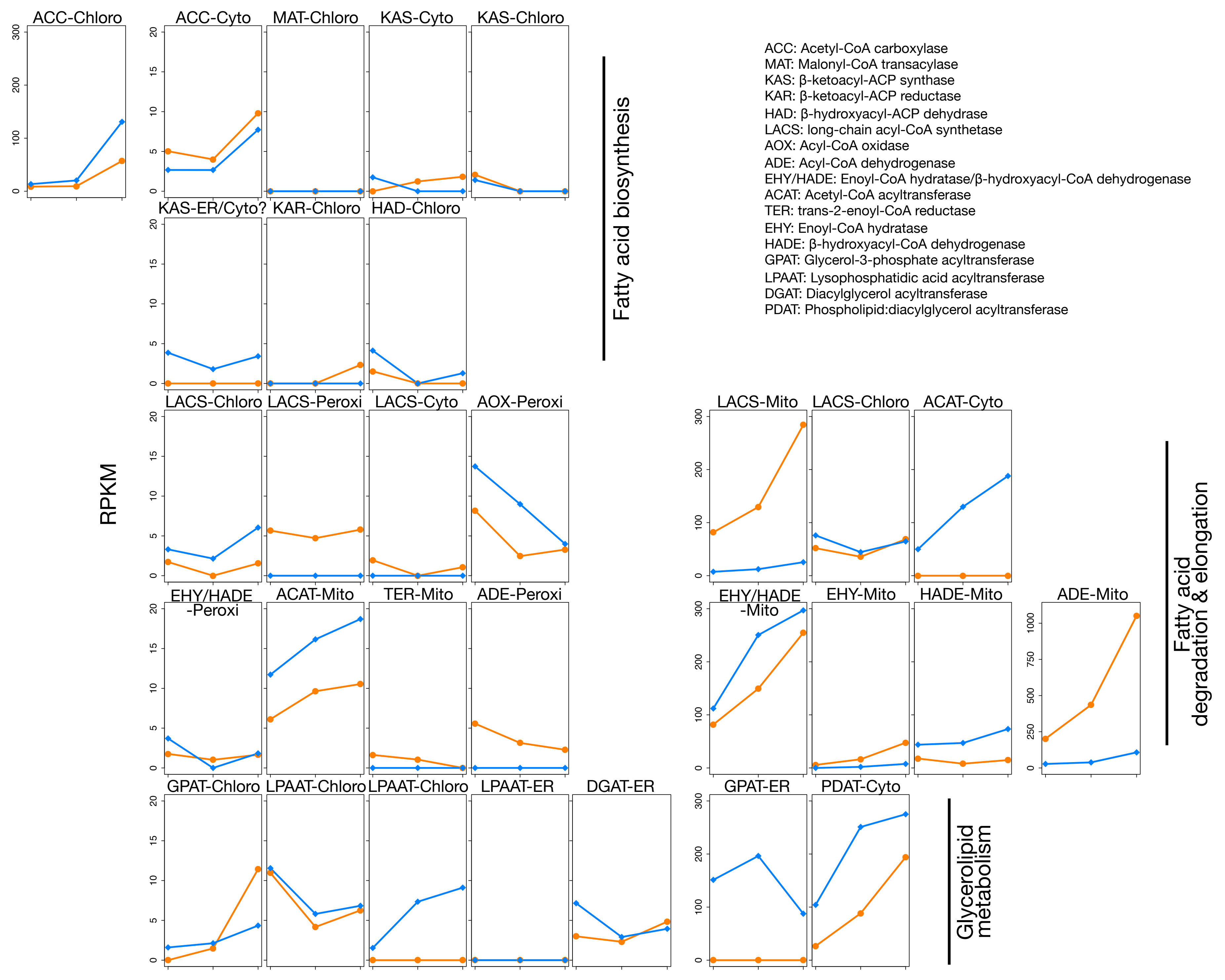
ACC-Chloro
ACC-Cyto
MAT-Chloro
KAS-Cyto
KAS-Chloro
KAS-ER/Cyto?
KAR-Chloro
HAD-Chloro
Fatty acid biosynthesis
LACS-Chloro
LACS-Peroxi
LACS-Cyto
AOX-Peroxi
LACS-Mito
LACS-Chloro
ACAT-Cyto
EHY/HADE -Peroxi
ACAT-Mito
TER-Mito
ADE-Peroxi
EHY/HADE -Mito
EHY-Mito
HADE-Mito
ADE-Mito
Fatty acid degradation & elongation
GPAT-Chloro
LPAAT-Chloro
LPAAT-Chloro
LPAAT-ER
DGAT-ER
GPAT-ER
PDAT-Cyto
Glycerolipid metabolism
RPKM
ACC: Acetyl-CoA carboxylase
MAT: Malonyl-CoA transacylase
KAS: β-ketoacyl-ACP synthase
KAR: β-ketoacyl-ACP reductase
HAD: β-hydroxyacyl-ACP dehydrase
LACS: long-chain acyl-CoA synthetase
AOX: Acyl-CoA oxidase
ADE: Acyl-CoA dehydrogenase
EHY/HADE: Enoyl-CoA hydratase/β-hydroxyacyl-CoA dehydrogenase
ACAT: Acetyl-CoA acyltransferase
TER: trans-2-enoyl-CoA reductase
EHY: Enoyl-CoA hydratase
HADE: β-hydroxyacyl-CoA dehydrogenase
GPAT: Glycerol-3-phosphate acyltransferase
LPAAT: Lysophosphatidic acid acyltransferase
DGAT: Diacylglycerol acyltransferase
PDAT: Phospholipid:diacylglycerol acyltransferase
